# Bioactive Metabolites from Mangrove Endophytic Fungus *Aspergillus* sp. 16-5B

**DOI:** 10.3390/md13053091

**Published:** 2015-05-19

**Authors:** Yayue Liu, Senhua Chen, Zhaoming Liu, Yongjun Lu, Guoping Xia, Hongju Liu, Lei He, Zhigang She

**Affiliations:** 1 School of Chemistry and Chemical Engineering, Sun Yat-Sen University, Guangzhou 510275, China; E-Mails: liuyayue@mail2.sysu.edu.cn (Y.L.); chensh65@mail2.sysu.edu.cn (S.C.); liuzhaom@mail2.sysu.edu.cn (Z.L.); xiagp@mail2.sysu.edu.cn (G.X.); liuhj8@mail2.sysu.edu.cn (H.L.); 2 School of Life Sciences and Biomedical Center, Sun Yat-Sen University, 135 Xin gang West Road, Guangzhou 510275, China; E-Mails: luyj@mail.sysu.edu.cn (Y.L.); helei8688@126.com (L.H.); 3 Key Laboratory of Functional Molecules from Oceanic Microorganism, Department of Education of Guangdong Province, Sun Yat-Sen University, Guangzhou 510080, China

**Keywords:** marine fungi, *Aspergillus* sp., α-glucosidase inhibitor, theoretical calculations, ECD, optical rotation

## Abstract

Chemical investigation of the endophytic fungus *Aspergillus* sp. 16-5B cultured on Czapek’s medium led to the isolation of four new metabolites, aspergifuranone (**1**), isocoumarin derivatives (±) **2** and (±) **3**, and (*R*)-3-demethylpurpurester A (**4**), together with the known purpurester B (**5**) and pestaphthalides A (**6**). Their structures were determined by analysis of 1D and 2D NMR spectroscopic data. The absolute configuration of Compound **1** was determined by comparison of the experimental and calculated electronic circular dichroism (ECD) spectra, and that of Compound **4** was revealed by comparing its optical rotation data and CD with those of the literature. The structure of Compound **6** was further confirmed by single-crystal X-ray diffraction experiment using CuKα radiation. All isolated compounds were evaluated for their α-glucosidase inhibitory activities, and Compound **1** showed significant inhibitory activity with IC_50_ value of 9.05 ± 0.60 μM. Kinetic analysis showed that Compound **1** was a noncompetitive inhibitor of α-glucosidase. Compounds **2** and **6** exhibited moderate inhibitory activities.

## 1. Introduction

Diabetes is a group of metabolic diseases in which there are high blood sugar levels over a prolonged period and can cause many serious complications, including diabetic ketoacidosis, non-ketotic hyperosmolar coma, cardiovascular disease, stroke, kidney failure, *etc*. [1,2,3]. The world Health Organization reported that there are 387 million people living with diabetes worldwide in 2014, and the number would increase to 592 million in 2035 [4]. Being a huge and growing global public health problem, diabetes dictates the urgent need to discover and develop new chemotherapeutic approaches to medical treatment.

**Figure 1:**
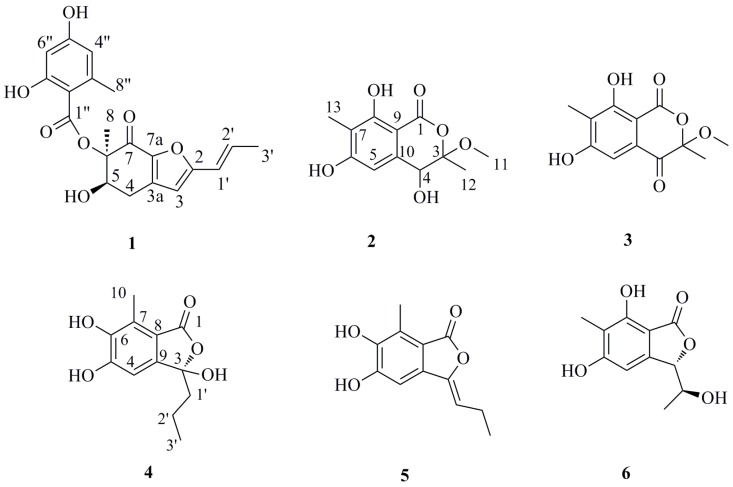
Chemical structures of Compounds **1**–**6**.

Endophytic fungi have been demonstrated to be a rich and reliable source of biologically-active and/or chemically-novel compounds [5,6,7]. In the past decade, our research group has focused on the exploration of new bioactive metabolites from mangrove endophytic fungi collected from the South China Sea [8,9,10,11], including some potential α-glucosidase inhibitors [12,13,14,15]. Recently, a chemical investigation of the mangrove-derived fungus *Aspergillus* sp. 16-5B, from the leaves of *Sonneratia apetala*, had led to the isolation and characterization of four new compounds (**1**–**4**), as well as two previously reported compounds (**5**,**6**) (Figure 1). All compounds were evaluated for their α-glucosidase inhibitory activities. Details of the isolation, structural elucidation, as well as evaluation of the biological activity of these compounds are reported herein.

## 2. Results and Discussion

**Figure 2:**
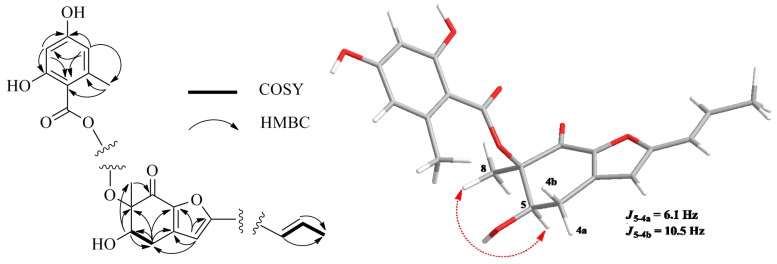
Selected ^1^H–^1^H COSY (bold line), HMBC (arrow) and key NOESY (dashed lines) correlations of Compound **1**.

Compound **1** was isolated as white amorphous powder. The molecular formula was deduced to be C_20_H_20_O_7_ (corresponding to eleven degrees of unsaturation) on the basis of its HREIMS molecular ion cluster at 372.1209 [M]^+^ (calcd. for C_20_H_20_O_7_, 372.1204). The IR spectrum showed absorption bands at 3394 and 1643 cm^−1^, which revealed the presence of hydroxyl and carbonyl groups. In the ^1^H NMR spectrum, the signals for two *meta*-coupled aromatic protons at δ_H_ 6.03 (d, *J* = 1.8 Hz, H-4′′) and 6.08 (d, *J* = 1.8 Hz, H-6′′), two *E*-configured olefinic protons at δ_H_ 6.28 (*J* = 15.8 Hz, H-1′) and 6.58 (*J* = 15.8 Hz, H-2′), one singlet olefinic proton (δ_H_ 6.16, H-3), one oxymethine (δ_H_ 5.11, H-5), one methylene (δ_H_ 3.07 and 2.70, H_2_-4) and three methyls (δ_H_ 1.56, H_3_-8; 2.39, H_3_-8′′; 1.83, H_3_-3′) were observed (Table 1). The ^1^H–^1^H COSY spectrum indicated two spin-systems of H-4/H-5 and H-1′/H-2′/H-3′, assigned to two fragments of –OCH–CH_2_– and –CH=CH–CH_3_ (Figure 2), respectively. The HMBC correlations from H_3_-8 to C-5, C-6 and C-7, from H-5 to C-4, C-6 and C-8, from H-4a to C-3, C-3a, C-5, C-6 and C-7a and from H-4b to C-5, C-3a and C-7a constructed a cyclohexenone fragment. The HMBC correlations from the singlet olefinic proton H-3 to C-1′, C-4, C-3a, C-7a and C-2 (δ_C_ 161.0) assembled a furan ring, which was connected with the cyclohexenone by sharing the bond of C-3a//C-7a. Judged by the following HMBC correlations of H-4′′ to C-3′′, C-5′′, C-6′′ and C-8′′; H-6′′ to C-2′′, C-4′′, C-5′′ and C-7′′ and H_3_-8′′ to C-2′′, C-3′′ and C-4′′, the moiety of orsellinic acid was constructed, which was connected to C-6 (δc 87.3) on the basis of an HMBC correlation from H_3_-8 to C-1′′. The HMBC correlations from H-1′ to C-2, C-3 and C-2′ determined that the fragment –CH=CH–CH_3_ was connected to C-2. Thus, the planar structure of **1** was deduced.

**Table 1:** ^13^C NMR (125 MHz) and ^1^H NMR (500 MHz) data of **1** (CDCl_3_).

Position	δc, Type	δ_H_, mult (*J* in Hz)
2	161.0, C	
3	107.8, CH	6.16, s
3a	138.7, C	
4	28.8, CH_2_	4a, 3.07, dd (16.6, 6.1)
		4b, 2.70, dd (16.6, 10.5)
5	69.6, CH	5.11, dd (6.1, 10.5)
6	87.3, C	
7	181.9, C	
7a	144.1, C	
8	16.4, CH_3_	1.56, s
1′	119.0, CH	6.28, dd (15.8, 1.4)
2′	134.2, CH	6.58, dd (15.8, 6.7)
3′	18.9, CH_3_	1.83, d (6.7)
1′′	170.3, C	
2′′	104.8, C	
3′′	144.2, C	
4′′	112.2, CH	6.03, d (1.8)
5′′	161.6, C	
6′′	101.2, CH	6.08, d (1.8)
7′′	164.9, C	
8′′	24.4, CH_3_	2.39, s

**Figure 3:**
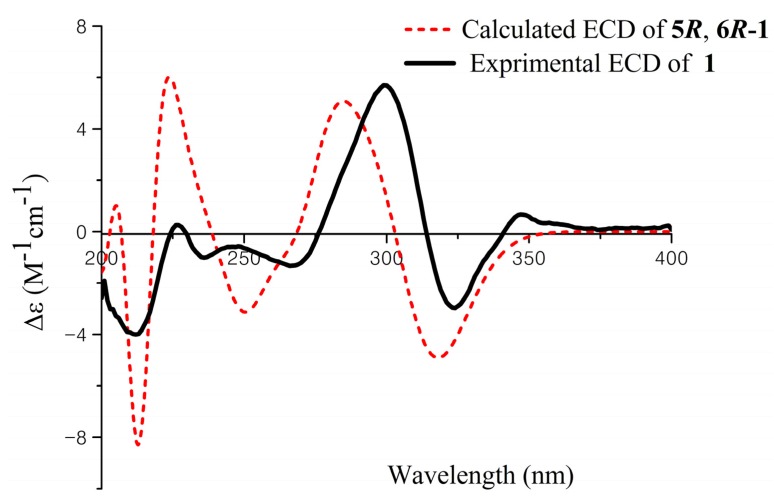
Experimental and calculated ECD spectra of **1**.

The relative configuration of **1** was established by interpretation of ^1^H NMR and NOESY data. In the ^1^H NMR spectrum, H-5 showed *ax*/*eq* coupling (*J* = 6.1 Hz) to H-4a and *ax*/*ax* coupling (*J* = 10.5 Hz) to H-4b. In the NOESY spectrum, the NOE correlation between H-5 and H_3_-8 as shown in Figure 2 supported the *syn* relationship of H-5 and H_3_-8. In order to establish the absolute configuration of **1**, the ECD spectrum of **1** was recorded and compared with density functional theory (DFT)-calculated spectra for the 5*R*, 6*R* and 5*S*, 6*S* isomers of **1** (Figure S25, Supplementary Information). The preliminary conformational distribution search was performed by Spartan 10 software using the MMFF94S force field. The corresponding minimum geometries were further fully optimized by DFT at the B3LYP/6-31G (d) level implemented in the Gaussian 03 program package. The obtained stable conformers were submitted to ECD calculation by the Time Dependent Density Functional Theory (TDDFT) cam-b3lyp/6-311+g (2d, p) method [16,17] (Figure S26, Supplementary Information). The calculated ECD spectra for isomer 5*R*, 6*R* showed a good fit with the experimental CD of **1**, which reproduced Cotton effects at 298 and 324 nm, respectively (Figure 3). Thus, the configuration of **1** was assigned to be 5*R*, 6*R*, named aspergifuranone.

**Figure 4:**
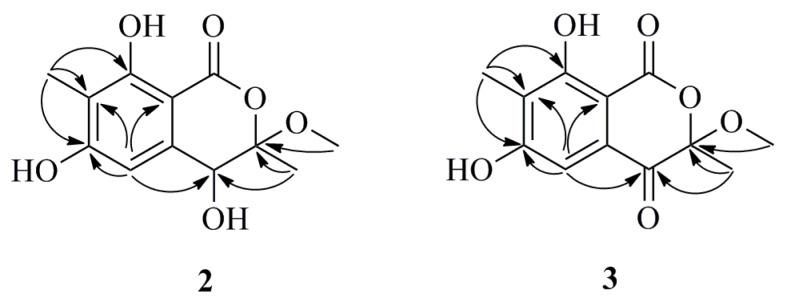
Selected HMBC (arrow) correlations of Compounds **2** and **3**.

Compound **2** was obtained as a white amorphous powder. Its molecular formula was deduced to be C_12_H_14_O_6_ on the basis of its HREIMS molecular ion cluster at *m*/*z* 254.0786 [M]^+^ (calcd. for C_12_H_14_O_6_, 254.0785). The IR spectrum displayed characteristic absorption bands at 3414 and 1661 cm^−1^, suggesting the presence of hydroxyl and carbonyl groups. The ^1^H and ^13^C NMR spectra of **2** revealed the signals of a penta-substituted aryl ring (δ_H_ 6.62, 1H, s; δ_C_ 162.9, 162.4, 141.2, 111.4, 107.8 and 100.0), a oxygenated methine (δ_H_ 4.46, 1H, s; δ_C_ 69.8), a methoxyl (δ_H_ 3.38, 3H, s; δ_C_ 50.4) and two methyls (δ_H_ 1.63, 3H, s; δ_C_ 18.4; δ_H_ 2.08, 3H, s; δ_C_ 7.9) (Table 2). In the HMBC spectrum, the correlations from 8-OH (δ_H_ 11.47) to C-7 and C-9, from H-5 to C-4, C-6, C-7 and C-9, from H_3_-13 (δ_H_ 2.08) to C-6, C-7 and C-8 and from H_3_-12 (δ_H_ 1.63) to C-3 and C-4 confirmed the existence of a 4,6,8-trihydroxy-3,7-dimethylisocoumarin unit (Figure 4). Moreover, the methoxyl group was attached to the C-3 position due to the HMBC correlation between H_3_-11 and C-3 (δ_C_ 108.3). Therefore, the planar structure of **2** was constructed as shown in Figure 4. The absence of any CD signal of **2** and the value of optical rotation was zero, all indicating a racemic mixture of the possible enantiomer. Subsequently, (±) it was attempted to separate **2** by chiral HPLC using three types of chiral columns, but all failed due to only one symmetrical peak in the chromatogram, as detected by HPLC.

Compound **3** was obtained as a white amorphous powder. Its molecular formula was determined to be C_12_H_12_O_6_ on the basis of its HREIMS molecular ion cluster at *m*/*z* 252.0630 [M]^+^ (calcd. for C_12_H_12_O_6_, 252.0628). Analysis of the ^1^H and ^13^C NMR data for **3** revealed the presence of nearly the same identical structural features as those found in **2**, except that the carbon signal of C-4 (δ_C_ 69.8) in **2** was absent and replaced by a carboxyl group signal (δ_C_ 190.1), which indicated that Compound **3** was the oxidative product of Compound **2** (Figure 4). However, the absence of any CD spectrum and zero optical rotation indicated that **3** was also a racemic mixture. Unfortunately, separation of **3**’s enantiomers also failed.

**Table 2:** ^1^H and ^13^C NMR data of Compounds **2** ^a^ and **3** ^b^.

Position	2		3	
	δc, Type	δ_H_, mult (*J* in Hz)	δc, Type	δ_H_, mult (*J* in Hz)
1	169.3, C		168.6, C	
2				
3	108.3, C		106.2, C	
4	69.8, CH	4.46, s	190.1, C	
5	107.8, CH	6.62, s	106.3, CH	7.01, s
6	162.9, C		164.1, C	
7	111.4, C		120.9, C	
8	162.4, C		162.6, C	
9	100.0, C		103.2, C	
10	141.2, C		130.6, C	
11	50.4, CH_3_	3.38, s	52.0, CH_3_	3.41, s
12	18.4, CH_3_	1.63, s	21.1, CH_3_	1.68, s
13	7.9, CH_3_	2.08, s	8.6, CH_3_	2.15, s
8-OH		11.47, s		

^a^ Data were recorded in acetone-*d*_6_ at 500 MHz for ^1^H NMR and 125 MHz for ^13^C NMR; ^b^ Data were recorded in methanol-*d*_4_ at 500 MHz for ^1^H NMR and 125 MHz for ^13^C NMR.

**Figure 5:**
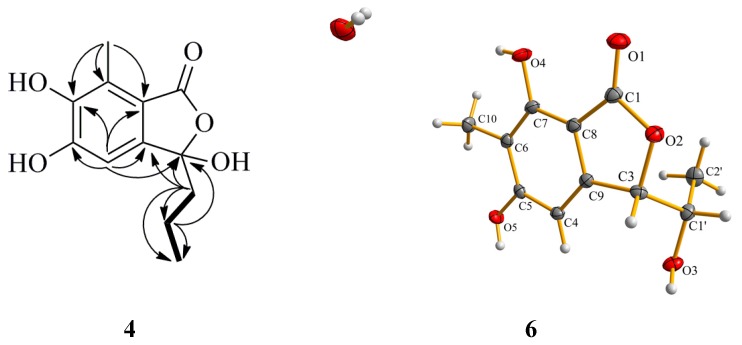
Selected HMBC (arrow) correlations of Compound **4** and perspective Oak Ridge Thermal Ellipsoid Plot (ORTEP) drawing for Compound **6**.

Compound **4** was obtained as a white amorphous powder. Its molecular formula was assigned to be C_12_H_14_O_5_ on the basis of its HREIMS molecular ion cluster at *m*/*z* 238.0835 [M]^+^ (calcd. for C_12_H_14_O_5_, 238.0836). The IR spectrum showed the presence of a hydroxyl group (3138 cm^−1^) and a conjugated carbonyl group (1741 cm^−1^). Overall inspections of the ^1^H and ^13^C NMR spectroscopic data revealed that Compound **4** also possessed a penta-substituted aryl ring (δ_H_ 6.59, 1H, s; δ_C_ 159.7, 152.2, 127.0, 125.7, 116.6 and 109.0) (Table 3). By analysis of the ^1^H–^1^H COSY spectrum, the correlation H_2_-1′/H_2_-2′ and H_2_-2′/H_3_-3′ indicated the presence of a propyl moiety. The HMBC correlations from H-4 to C-3 (δ_C_ 107.8), C-5 (δ_C_ 152.2), C-6 (δ_C_ 159.7), C-8 (δ_C_ 116.6) and C-9 (δ_C_ 127.0) and from H_3_-10 to C-6 (δ_C_ 159.7), C-7 (δ_C_ 125.7) and C-8 (δ_C_ 116.6) indicated the position of two hydroxyl group were ortho. The correlations from H_3_-10 to C-7 (δ_C_ 125.7) and from H-1′ to C-3 (δ_C_ 107.8) revealed that the methyl and propyl groups were attached to the C-7 and C-3 positions, respectively (Figure 5). Thus, the planar structure of Compound **4** was constructed. The absolute configuration of **4** was established by comparing its optical rotation data and CD with those of the literature [18]. The opposite CD Cotton effects to purpurester A at 286 nm (∆ɛ_max_ + 0.8) and 232 nm (∆ɛ_max_ − 1.5) and the opposite optical direction of ${\left[\alpha \right]}_{D}^{25}$ +30 (*c* 0.1, MeOH) clearly revealed the 3*R*-configuration*,* named (*R*)-3-demethylpurpurester A.

**Table 3:** ^13^C (125 MHz) and ^1^H (500 MHz) NMR data of Compounds **4** and **5** (methanol-*d*_4_).

Position	4		5	
	δc, Type	δ_H_, mult (*J* in Hz)	δc, Type	δ_H_, mult (*J* in Hz)
1	171.6, C		167.5, C	
2				
3	107.8, C		143.7, C	
4	109.0, CH	6.59, s	108.6, CH	6.64, s
5	152.2, C		151.6, C	
6	159.7, C		158.1, C	
7	125.7, C		123.7, C	
8	116.6, C		114.9, C	
9	127.0, C		117.3, C	
10	9.4, CH_3_	2.35, s	9.8, CH_3_	2.37, s
1′	40.3, CH_2_	2.18, m	109.9, CH	5.69, t (7.7)
2′	18.1, CH_2_	1.26, m	19.5, CH_2_	2.40, m
		1.06, m		
3′	14.3, CH_3_	0.88, t (7.4)	14.8, CH_3_	1.11, t (7.7)

The structures of the known compounds were identified as purpuresters B (**5**) [18] (Table 3) and pestaphthalides A (**6**) [19] by comparison of their spectroscopic and optical rotation data with those reported in the literature. The structure of Compound **6** was further confirmed by single-crystal X-ray diffraction experiment using CuKα radiation [20] (Figure 5) (Cambridge Crystallographic Data Centre (CCDC) Number 1053019).

**Figure 6:**
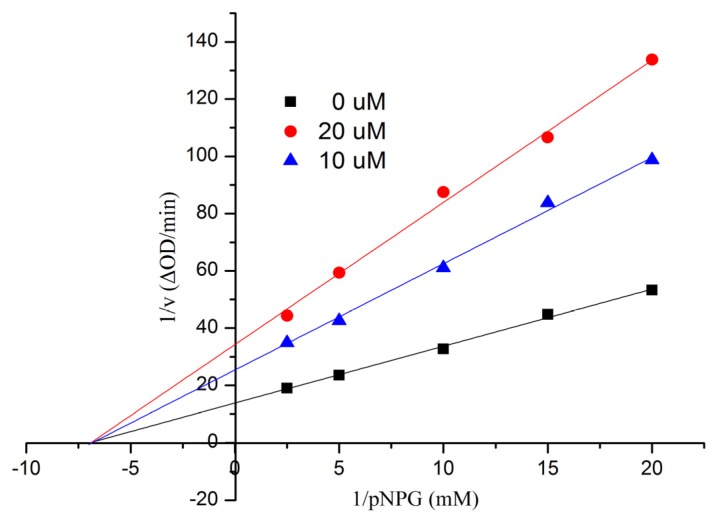
Kinetic analysis of the inhibition of α-glucosidase of **1**.

All compounds were tested for their *in vitro* inhibitory activities against α-glucosidase along with the clinical α-glucosidase inhibitor acarbose (positive control) [21]. As a result (Table 4), Compound **1** was the most active and showed better inhibitory potential (IC_50_ = 9.05 ± 0.60 μM) than acarbose (IC_50_ = 553.7 ± 6.8 μM). In order to examine the inhibition type of Compound **1**, further kinetic studies were carried out by the Lineweaver-Burk plot method. The results are shown in Figure 6, indicating that **1** was a noncompetitive inhibitor of α-glucosidase. Compounds **2** and **6** exhibited more efficacy than that of the positive control, with IC_50_ values of 90.4 ± 2.9 and 69.6 ± 3.5 μM, respectively. More details are given in the Supplementary Information.

**Table 4:** Inhibitory effects of the isolates against α-glucosidase ^a^.

Compound	1	2	3	4	5	6	Acarbose ^b^
IC_50_ (μM)	9.05 ± 0.60	90.4 ± 2.9	>200	>200	>200	69.6 ± 3.5	553.7 ± 6.8

^a^ IC_50_ values are shown as the mean ± SD from three independent experiments; ^b^ Positive control.

## 3. Experimental Section

### 3.1. General

Melting points were measured on an X-4 micro-melting-point apparatus (Cany Precision Instruments Co., Ltd., Shanghai, China) and are uncorrected. Optical rotations were recorded with an MCP 300 (Anton Paar, Shanghai, China) polarimeter at 25 °C. UV data were measured on a UV-240 spectrophotometer (Shimadzu, Beijing, China). IR spectra were recorded on a Nicolet Nexus 670 spectrophotometer (Thermo Fisher Scientific, Inc., Hudson, NH, USA) in KBr discs. CD data were measured on a Chirascan™ CD spectrometer (Applied Photophysics, London, UK). ^1^H and ^13^C NMR spectra were recorded on a Bruker AVANCE 500 spectrometer (Bruker BioSpin Corporation, Bellerica, MA, USA) in CDCl_3_, acetone-*d*_6_ or methanol-*d*_4_. Chemical shifts were reported in δ (ppm), using Tetramethylsilane (TMS) as the internal standard, and coupling constants (*J*) were reported in Hertz (Hz). EIMS spectra were measured on a Thermo DSQ EIMS spectrometer and HREIMS on a Thermo MAT95XP high-resolution mass spectrometer. Single-crystal data were measured on an Agilent Gemini Ultra diffractometer (CuKα radiation). Column chromatography (CC) was performed using silica gel (200–300 mesh, Qingdao Huang Hai Chemical Group Co., Qingdao, China; G60, F-254) and Sephadex LH-20 (GE Healthcare, Buckinghamshire, UK) stationery phases.

### 3.2. Fungal Material

The mangrove endophytic fungal strain 16-5B used in this study was isolated from the leaves of *Sonneratia apetala*, which was collected from Dongzhaigang Mangrove National Nature Reserve in Hainan Island, China, in April, 2009. The strain was identified by Yayue Liu as *Aspergillus* sp., according to morphologic traits and molecular identification. Its 444 base pair internal transcribed spacer (ITS) sequence had 99% sequence identity to that of *Aspergillus* sp. (No. JF312217). The sequence data had been submitted to GenBank with Accession Number KP059102. A voucher specimen (Registration Number 16-5B) has been deposited at the School of Chemistry and Chemical Engineering, Sun Yat-Sen University, Guangzhou, China.

### 3.3. Extraction and Isolation

The fungus *Aspergillus* sp. was cultivated in Czapek’s medium (3 g NaNO_3_, 1 g K_2_HPO_4_, 0.5 g MgSO_4_, 1 g KCl, 0.01 g FeSO4, 20 g sucrose and 20 g agar in 1 L water) in 1-L Erlenmeyer flasks, each containing 300 mL of culture broth at 25 °C without shaking for 28 days. The culture (40 L) was filtered to separate the mycelia from culture broth, and then, the mycelia were extracted with MeOH three times. The organic solvent was filtered and concentrated under reduced pressure to yield 6.9 g of organic extract, which was subjected to silica gel CC using gradient elution with petroleum ether-EtOAc from 90:10 to 0:100 (*v*/*v*) to give nine fractions (Fractions 1–9). Fraction 3 was further purified by silica gel CC using 40% EtOAc-petroleum ether to afford seven subfractions (Fractions 3.1–3.7). Fraction 3.3 was applied to Sephadex LH-20 CC, eluted with MeOH to obtain Compound **1** (3.2 mg). Fraction 6 was applied to Sephadex LH-20 CC, eluted with CHCl_3_/MeOH (1:1) to afford five subfractions (Fractions 6.1–6.5). Fraction 6.3 was further purified with Sephadex LH-20 CC, eluted with MeOH to obtain Compound **2** (1.5 mg). Fraction 2 was further purified by silica gel CC using 1% MeOH/CHCl_3_ to obtain Compound **3** (2.1 mg). Fraction 4 was further purified by silica gel CC using gradient elution with MeOH/CHCl_3_ from 1:99–1:9 (*v*/*v*) to afford five subfractions (Fractions 4.1–4.5). Fraction 4.2 was further purified using 3% MeOH/CHCl_3_ to obtain Compound **5** (5.8 mg). Fraction 4.4 was further purified by Sephadex LH-20 CC eluted with CHCl_3_/MeOH (1:1) to obtain Compound **4** (5.8 mg). Compound **6** (20.5 mg) was obtained by recrystallization with acetone from Fraction 5.

Compound **1**: white amorphous powder; mp 121.1–121.6 °C; ${\left[\alpha \right]}_{D}^{25}$ –110 (*c* 0.1, MeOH); UV (MeOH) λ_max_: 319 (3.33), 268 (3.11) and 217 (3.55) nm; CD (CH_3_OH) λ_max_ (∆ɛ) 324 (–3.1), 298 (+3.6), 267 (–1.3), 221 (+0.3), 211 (–4.0) nm; IR (KBr) *υ*_max_ 3394, 2933, 2851, 1643, 1505, 1450, 1380, 1321, 1266, 1171, 1079, 998 and 847 cm^−1^; ^1^H NMR (CDCl_3_, 500 MHz) and ^13^C NMR (CDCl_3_, 125 MHz), see Table 1; EIMS *m*/*z* 372 [M]^+^; HREIMS *m*/*z* 372.1209 [M]^+^ (calcd. for C_20_H_20_O_7_, 372.1204)

Compound **2**: white amorphous powder; mp 174.4–174.9 °C, ${\left[\alpha \right]}_{D}^{25}$ 0 (*c* 0.1, MeOH); UV (MeOH) λ_max_: 309 (2.81), 277 (3.21) and 218 (3.50) nm; IR (KBr) *υ*_max_ 3365, 2922, 1653, 1510, 1450, 1330, 1206, 1101, 1041 and 836 cm^−1^; ^1^H NMR (acetone-*d*_6_, 500 MHz) and ^13^C NMR (acetone-*d*_6_, 125 MHz), see Table 2; EIMS 254 [M]^+^; HREIMS *m*/*z* 254.0786 [M]^+^ (calcd. for C_12_H_14_O_6_, 254.0785).

Compound **3**: white amorphous powder; mp 134.2–134.8 °C; ${\left[\alpha \right]}_{D}^{25}$ 0 (*c* 0.1, MeOH); UV(MeOH) λ_max_: 342 (2.72), 319 (2.86) and 251 (3.47) nm; IR (KBr) *υ*_max_ 3239, 2922, 1721, 1627, 1309, 1189, 1085, 1037 and 870 cm^−1^; ^1^H NMR (methanol-*d*_4_, 500 MHz) and ^13^C NMR (methanol-*d*_4_, 125 MHz), see Table 2; EIMS *m*/*z* 252 [M]^+^; HREIMS *m*/*z* 252.0630 [M]^+^ (calcd. for C_12_H_12_O_6_, 252.0628).

Compound **4**: white amorphous powder; mp 201.2–201.9 °C ${\left[\alpha \right]}_{D}^{25}$ +30 (*c* 0.1, MeOH); UV(MeOH) λ_max_: 326 (2.68), 258 (2.76) and 209 (3.59) nm; CD (CH_3_OH) λ_max_ (∆ɛ) 329 (+0.2), 286 (+0.8), 232 (–1.5) nm; IR (KBr) *υ*_max_ 3601, 3311, 3151, 2961, 1734, 1623, 1521, 1460, 1394, 1343, 1279, 1208, 1098 and 864 cm^−1^; ^1^H NMR (methanol-*d*_4_, 500 MHz) and ^13^C NMR (methanol-*d*_4_, 125 MHz), see Table 3; EIMS *m*/*z* 238 [M]^+^; HREIMS *m*/*z* 238.0835 [M]^+^ (calcd. for C_12_H_14_O_5_, 238.0836).

### 3.4. Assay for α-Glucosidase Inhibitory Activity

All of the tests for α-glucosidase inhibitory activity were according to a previously described method [22,23]. The reaction mixture (final volume, 1 mL) consisted of the enzyme solution (20 μL, Sigma 9001-42-7, E.C 3.2.1.20), substrate (10 mM *p*-nitrophenyl-α-glucopyranoside, 20 μL, Fluka, BioChemika, Buchs, Switzerland) in 50 mM potassium phosphate buffer (pH 7.0) and 20 μL DMSO or inhibitor (test sample dissolved in DMSO (10 μmol/mL)). The inhibitors were pre-incubated with the enzyme at 37 °C for 20 min, and the substrate was then added. The reaction was monitored spectrophotometrically by measuring the absorbance at 400 nm for 1-min intervals. Calculations were performed according to the equation: inhibition rates (%) = ((OD_control_ − OD_control blank_) − (OD_test_ − OD_test blank_))/(OD_control_ − OD_control blank_) × 100%. The IC_50_ values of the compounds were calculated by nonlinear regression analysis and expressed as the mean ± SD of two distinct experiments. Kinetic parameters were determined using the Lineweaver-Burk double-reciprocal plot method at increasing concentrations of substrates and inhibitors.

### 3.5. Quantum Mechanical Calculation

In theoretical calculations, the preliminary conformational distribution search was performed by Spartan’10 software (Wavefunction, Inc., Irvine, CA, USA) using the MMFF94S force field. The corresponding minimum geometries were further fully optimized with the Gaussian 03 (Gaussian, Wallingford, CT, USA) program package at the B3LYP/6-31G (d) computational level as frequency calculations. The obtained stable conformers were submitted to CD calculation by the TDDFT cam-b3lyp/6-311+g (2d, p) method. ECD spectra were generated using the program SpecDis 1.6 (University of Würzburg, Würzburg, Germany) and OriginPro 8.5 (OriginLab, Ltd., Northampton, MA, USA) from dipole-length rotational strengths by applying Gaussian band shapes with sigma = 0.19 electron volt (ev). All calculations were performed with the High-Performance Grid Computing Platform of Sun Yat-Sen University.

## 4. Conclusions

Chemical investigation of *Aspergillus* sp. 16-5B, a marine endophytic fungus isolated from the leaves of *Sonneratia apetala*, led to the discovery of six compounds (**1**–**6**), including new compound aspergifuranone (**1**), two new pairs of enantiomers of isocoumarin derivatives (±) **2** and (±) **3**, and (*R*)-3-demethylpurpurester A (**4**). The structures of the isolates were established by 1D and 2D NMR spectroscopic data, as well as ECD calculation and optical rotation data. Meanwhile, the previously reported Compound **6** was obtained as a suitable crystal and confirmed by single-crystal X-ray diffraction experiment using CuKα radiation for the first time. All isolated compounds were evaluated for their α-glucosidase inhibitory effects. Compounds **1**, **2** and **6** exhibited more potent inhibitory effects against α-glucosidase activity than the clinical *α*-glucosidase inhibitor acarbose. Meanwhile, further mechanistic analysis showed that Compound **1** exhibited noncompetitive inhibition characteristics.

## Supplementary Files

Supplementary File 1
