# Supplementary material for: Bioactive Metabolites from Mangrove Endophytic Fungus Aspergillus sp. 16-5B
Source: Mar Drugs. 2015 May 19;13(5):3091–102. doi: 10.3390/md13053091 (PMC4446620; doi:10.3390/md13053091)
Supplement: Supplementary File 1 [file marinedrugs-13-03091-s001.pdf]

Supplementary Information

Contents	page
**Figure S1.** HREIMS spectrum of Compound **1**	S2
**Figure S2.** 1H NMR (500 MHz, CDCl3) spectrum of Compound **1**	S2
**Figure S3.** 13C NMR (125MHz, CDCl3) spectrum of Compound **1**	S3
**Figure S4.** ^{1}H–^{1}H COSY spectrum of Compound **1**	S3
**Figure S5.** HSQC spectrum of Compound **1**	S4
**Figure S6.** HMBC spectrum of Compound **1**	S4
**Figure S7.** NOESY spectrum of Compound **1**	S5
**Figure S8.** HREIMS spectrum of Compound **2**	S5
**Figure S9.** 1H NMR (500 MHz, acetone-d_6) spectrum of Compound **2**	S6
**Figure S10.** 13C NMR (125MHz, acetone-d_6) spectrum of Compound **2**	S6
**Figure S11.** HSQC spectrum of Compound **2**	S7
**Figure S12.** HMBC spectrum of Compound **2**	S7
**Figure S13.** NOESY spectrum of Compound **2**	S8
**Figure S14.** HREIMS spectrum of Compound **3**	S8
**Figure S15.** 1H NMR (500 MHz, methanol-d_4) spectrum of Compound **3**	S9
**Figure S16.** ^{13}C NMR (125 MHz, Methanol-d_4) (**A**) and DEPT spectra of Compound **3** (**B**)	S10
**Figure S17.** HSQC spectrum of Compound **3**	S11
**Figure S18.** HMBC spectrum of Compound **3**	S11
**Figure S19.** HREIMS spectrum of Compound **4**	S12
**Figure S20.** 1H NMR (500 MHz, methanol-d_4) spectrum of Compound **4**	S12
**Figure S21.** ^{13}C NMR (125 MHz, Methanol-d_4) (**A**) and DEPT spectra of Compound **4** (**B**)	S13
**Figure S22.** 1H–1H COSY spectrum of Compound **4**	S14
**Figure S23.** HSQC spectrum of Compound **4**	S14
**Figure S24.** HMBC spectrum of Compound **4**	S15
**Figure S25.** The structures (**A**) and calculated ECD spectra of the two isomers of 5*R*,6*R*-**1** (**B**) and 5*S*,6*S*-**1** (**C**).	S15
**Figure S26.** DFT-optimized low-energy structures (**1-1**–**1-5**) for 5*R*,6*R*	S17
**Figure S27.** CD spectrum of Compound **4**	S18

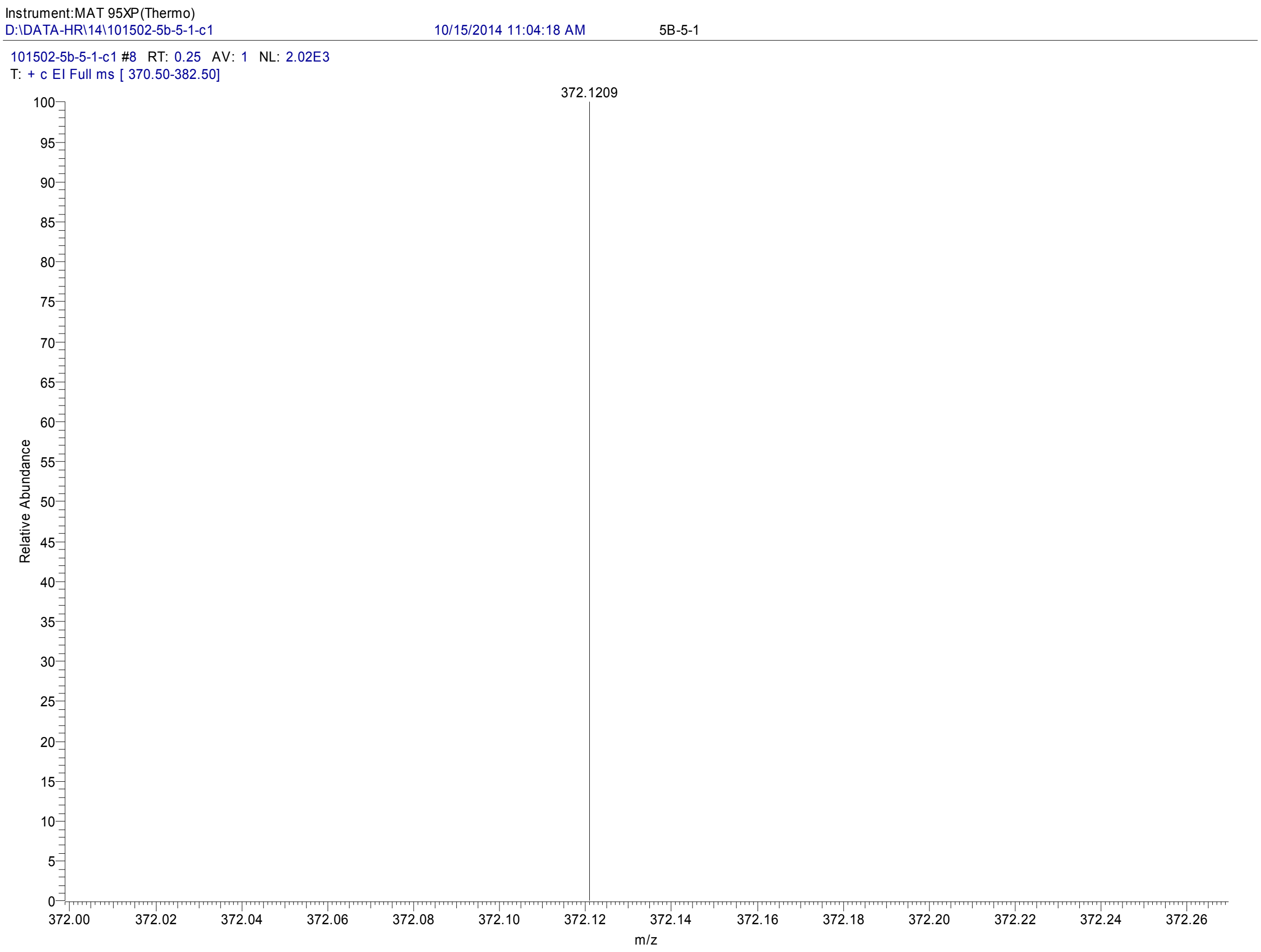

Figure S1. HREIMS spectrum of Compound **1**.

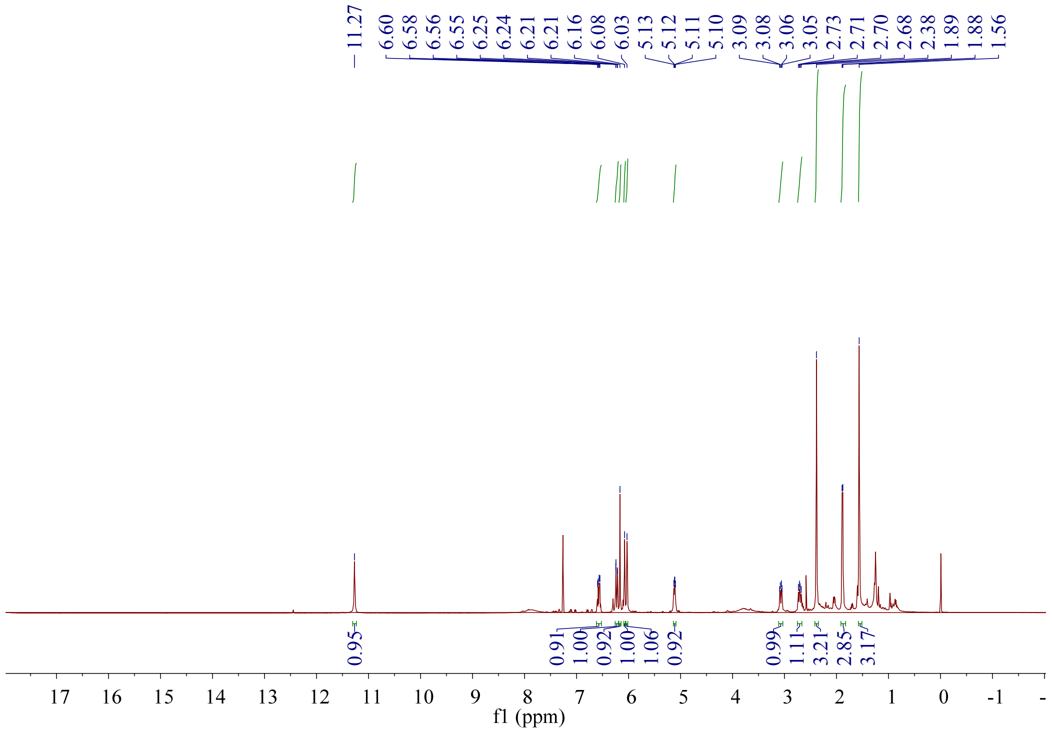

Figure S2. ^{1}H NMR (500 MHz, $CDCl_3$) spectrum of Compound **1**.

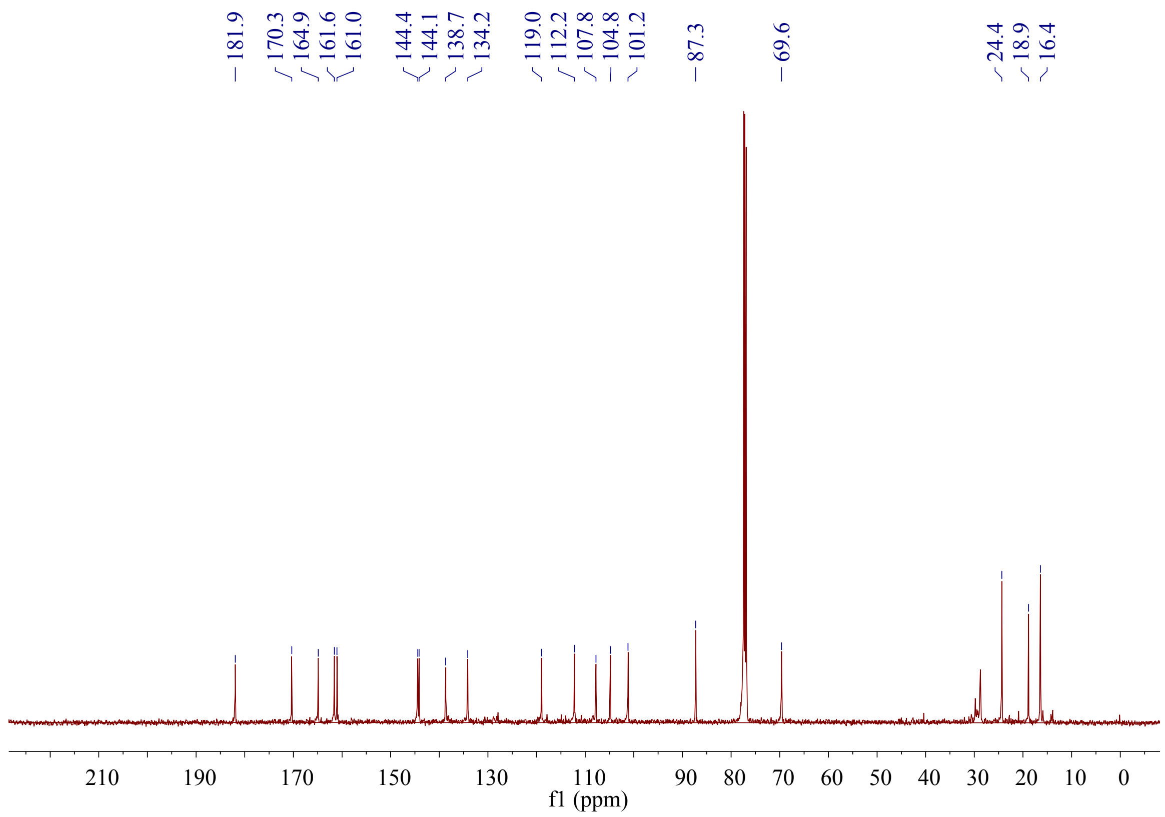

Figure S3. ^{13}C NMR (125 MHz, CDCl3) spectrum of Compound **1**.

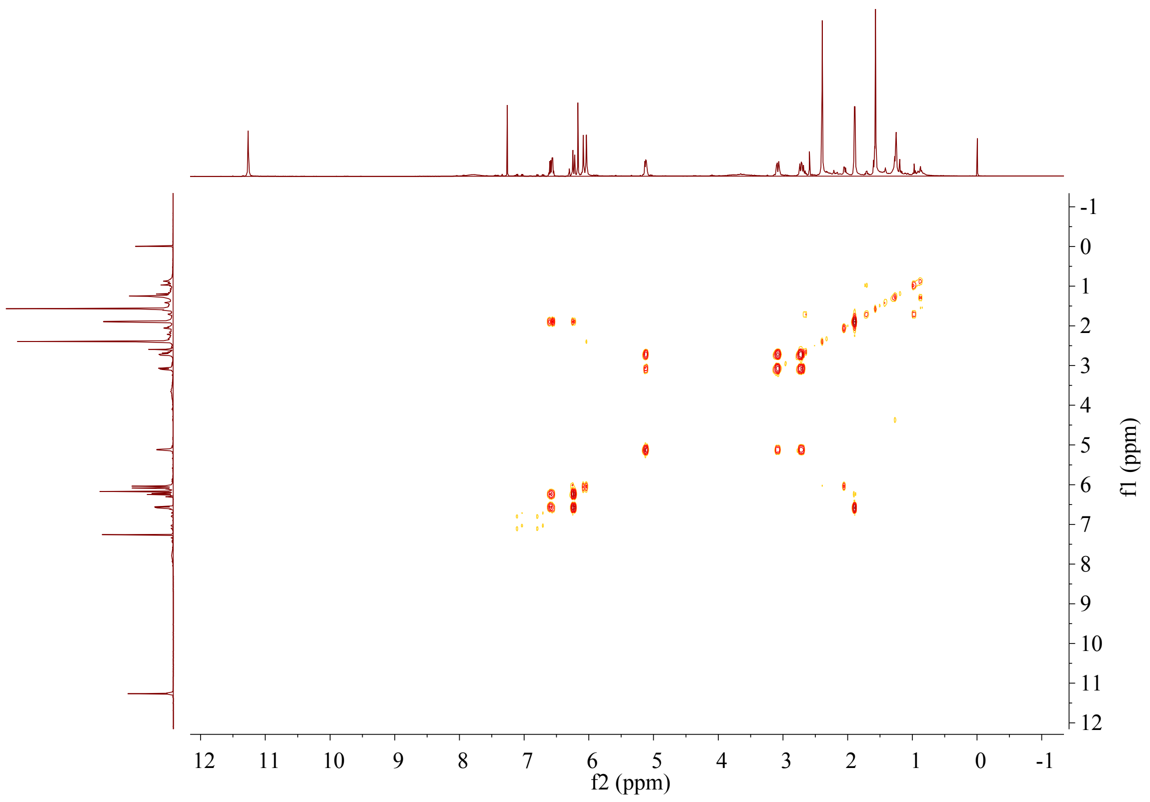

Figure S4. ^{1}H–^{1}H COSY spectrum of Compound **1**.

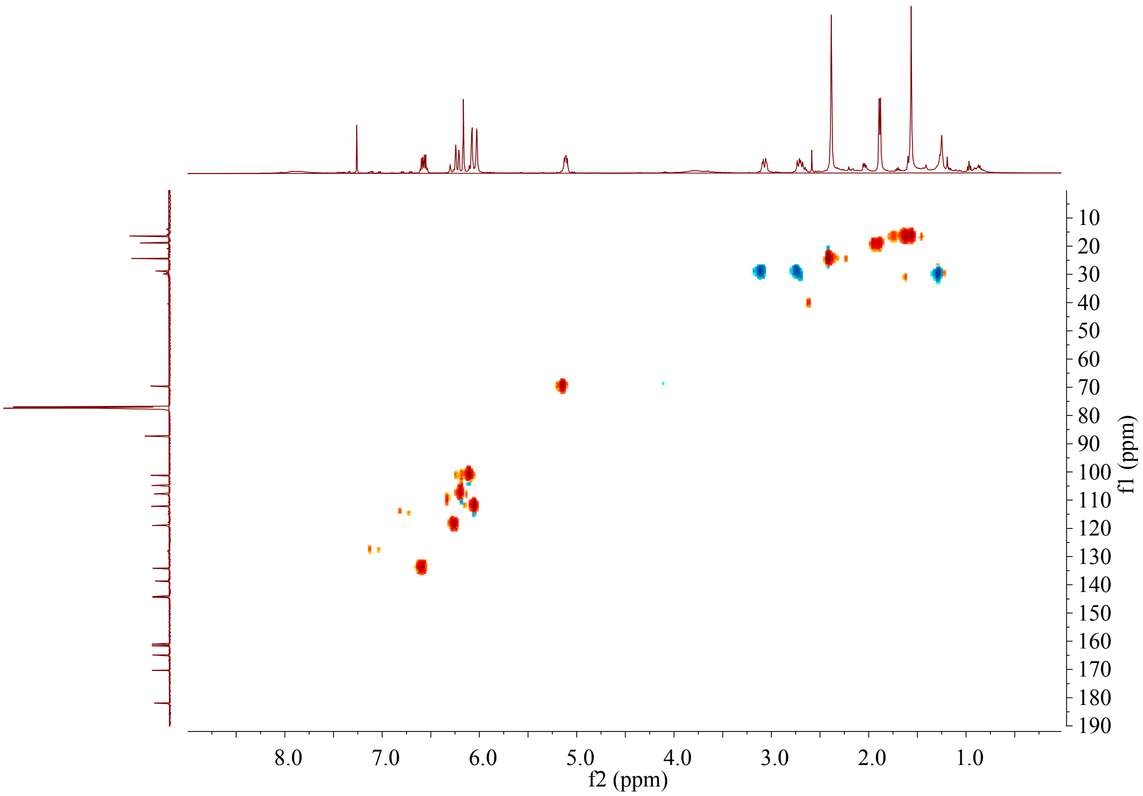

Figure S5. HSQC spectrum of Compound **1**.

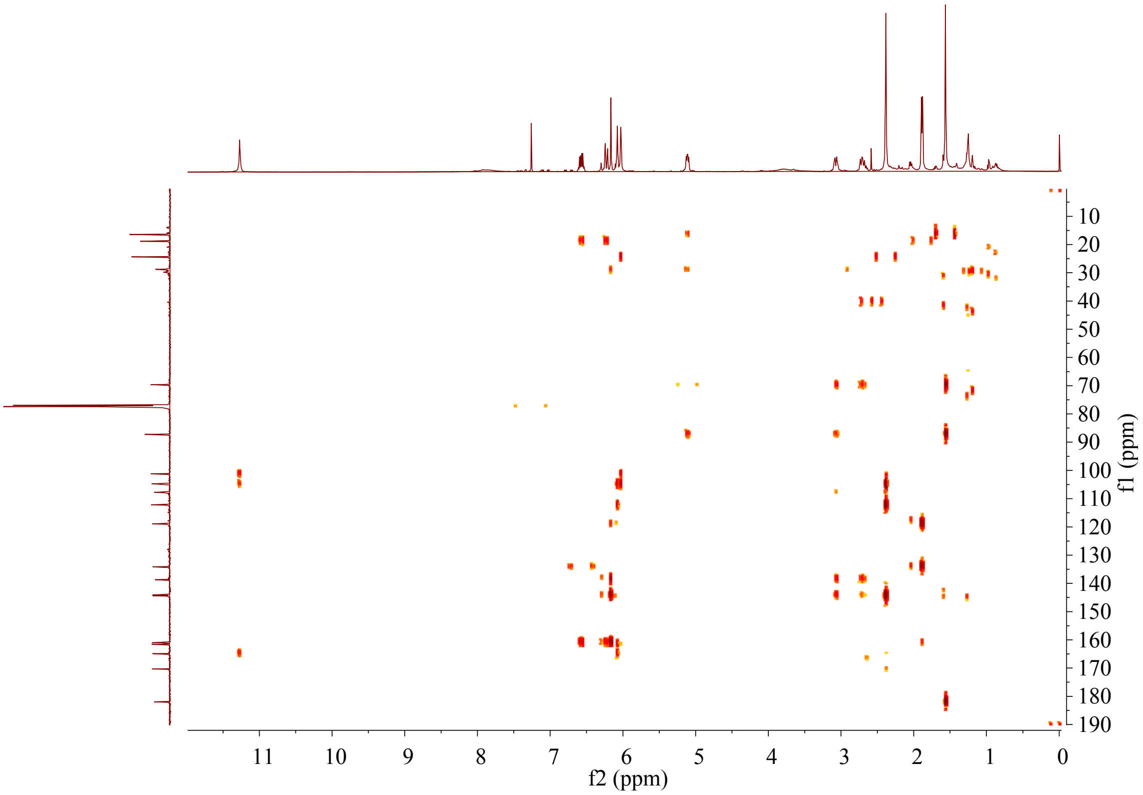

Figure S6. HMBC spectrum of Compound **1**.

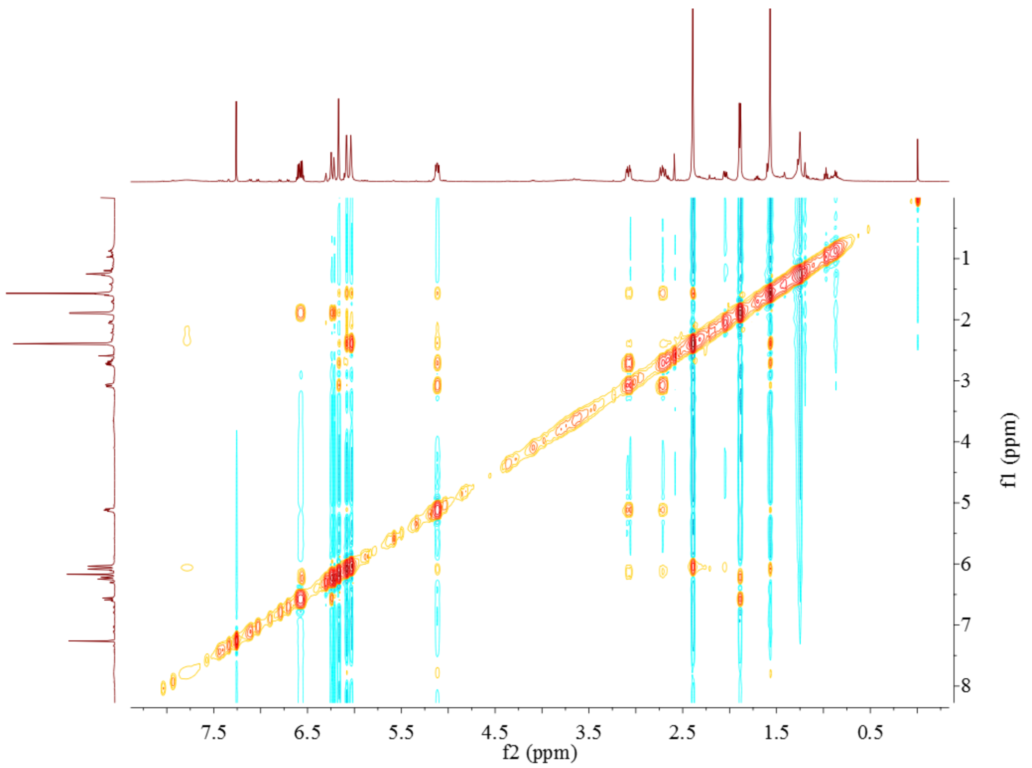

Figure S7. NOESY spectrum of Compound **1**.

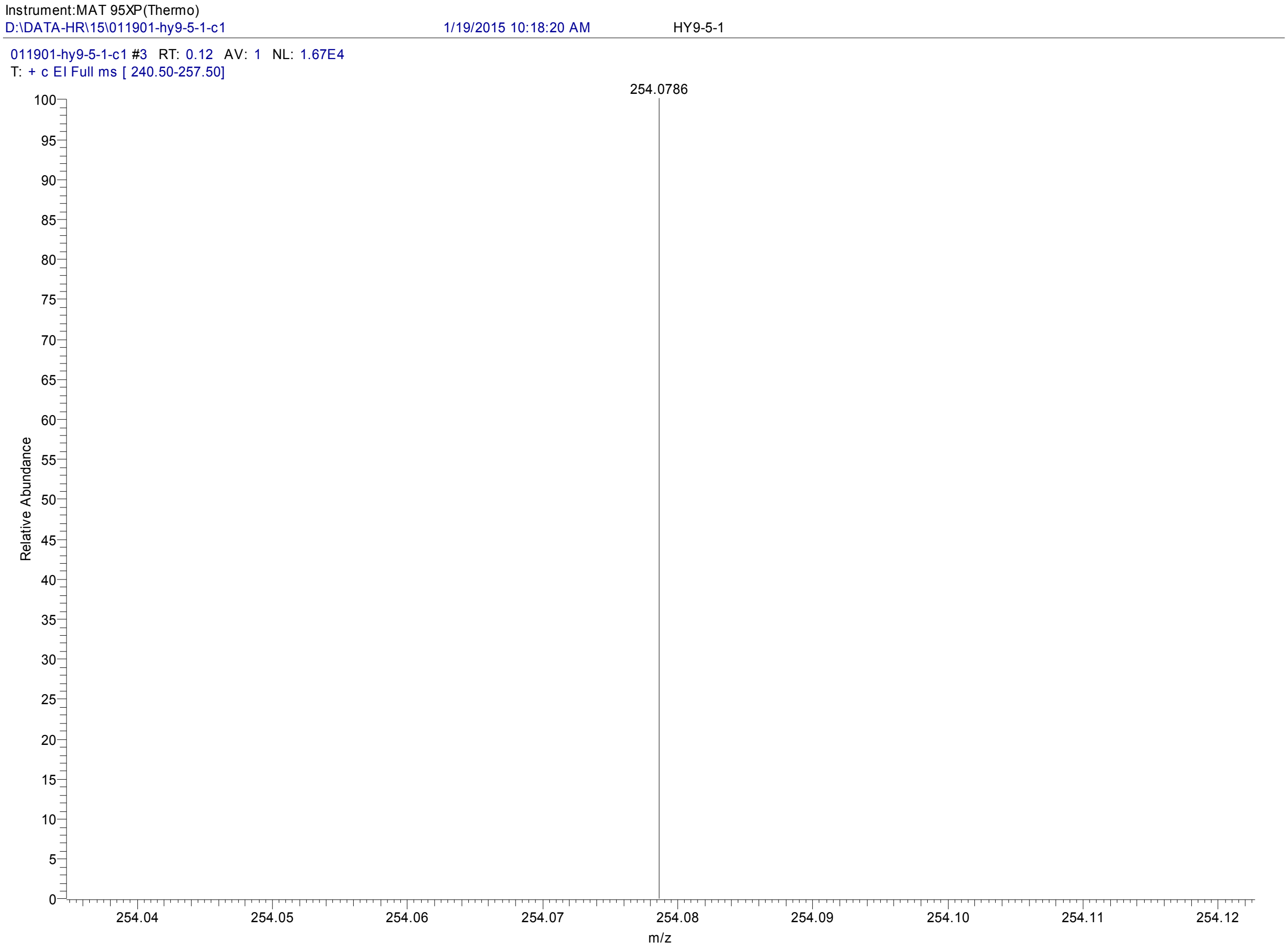

Figure S8. HREIMS spectrum of Compound **2**.

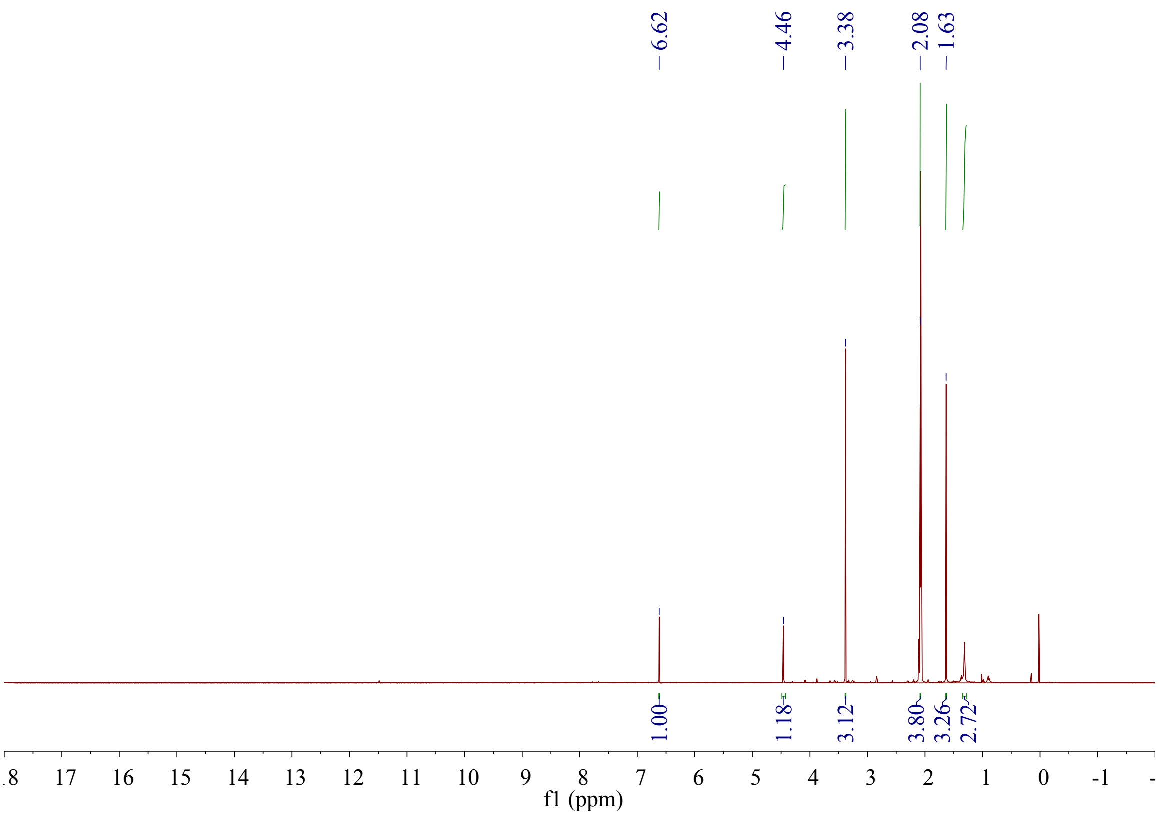

Figure S9. ^{1}H NMR (500 MHz, acetone-d_6) spectrum of Compound **2**.

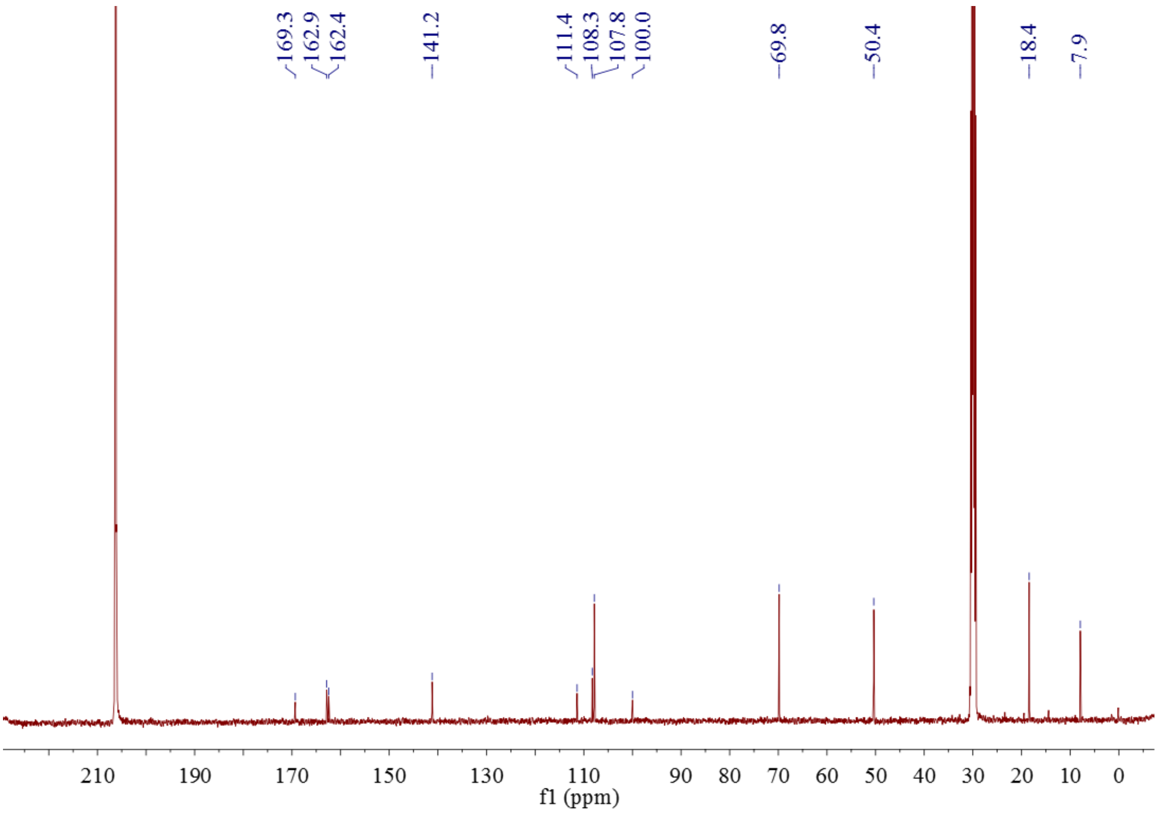

Figure S10. ^{13}C NMR (125 MHz, acetone-d_6) spectrum of Compound **2**.

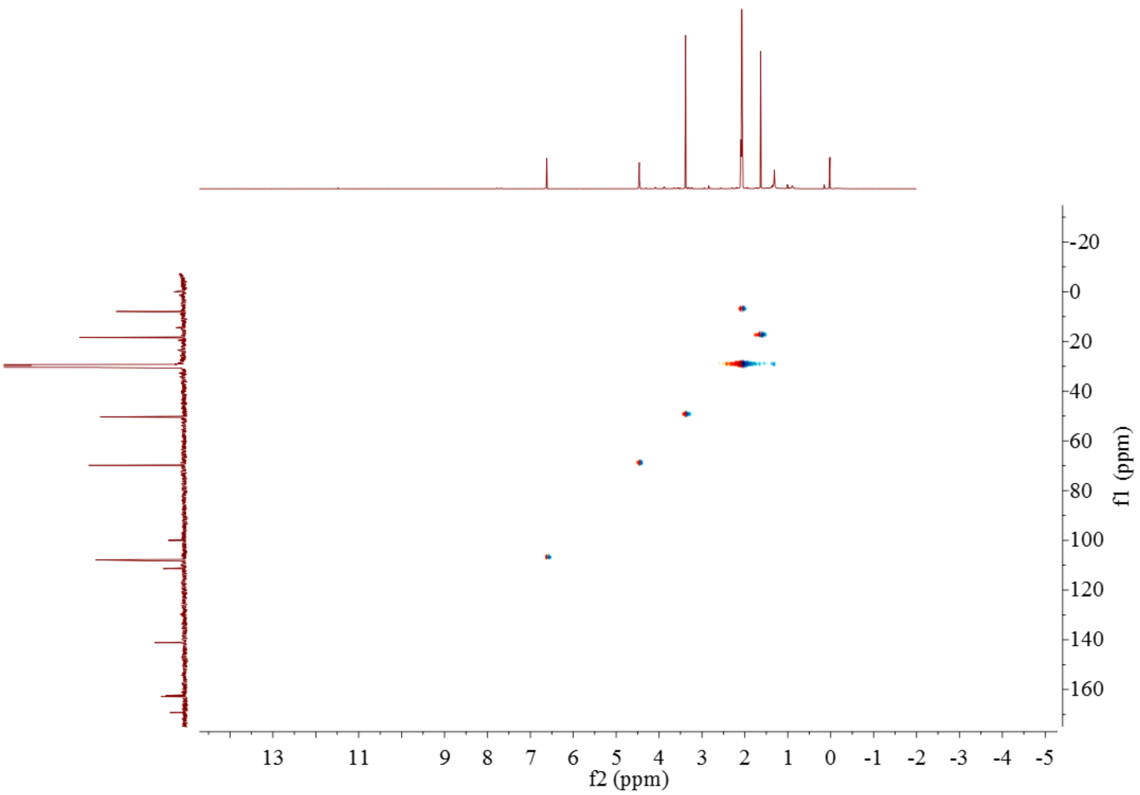

Figure S11. HSQC spectrum of Compound **2**.

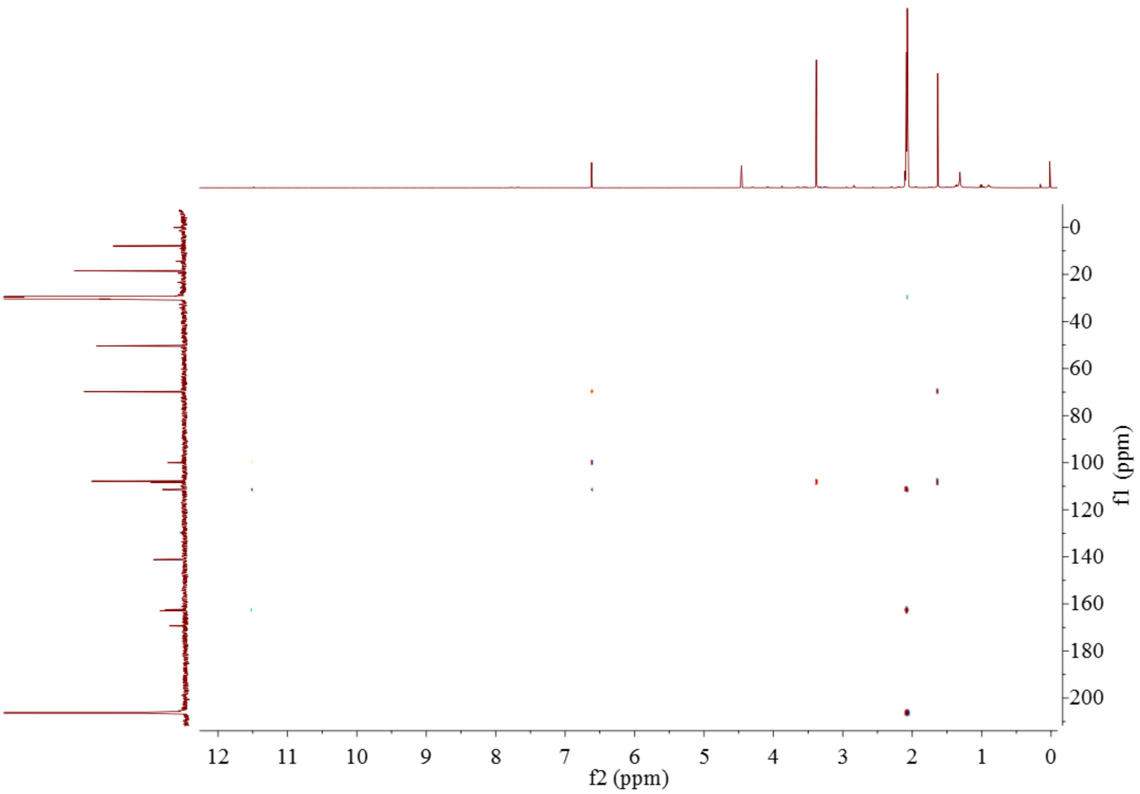

Figure S12. HMBC spectrum of Compound **2**.

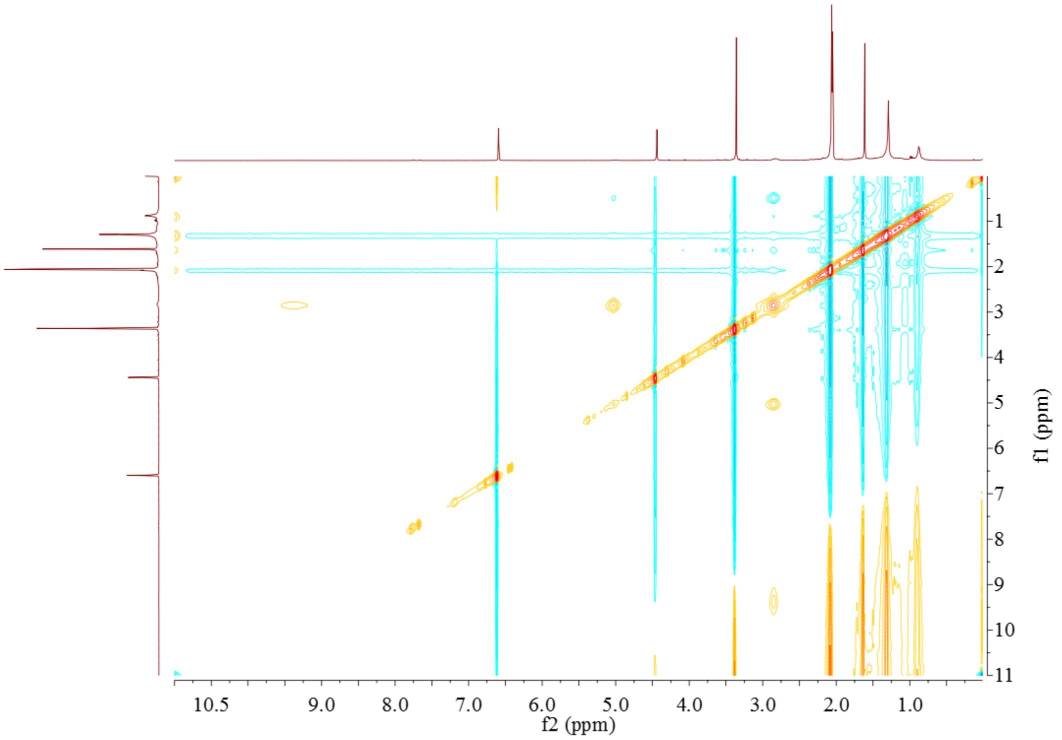

Figure S13. NOESY spectrum of Compound **2**.

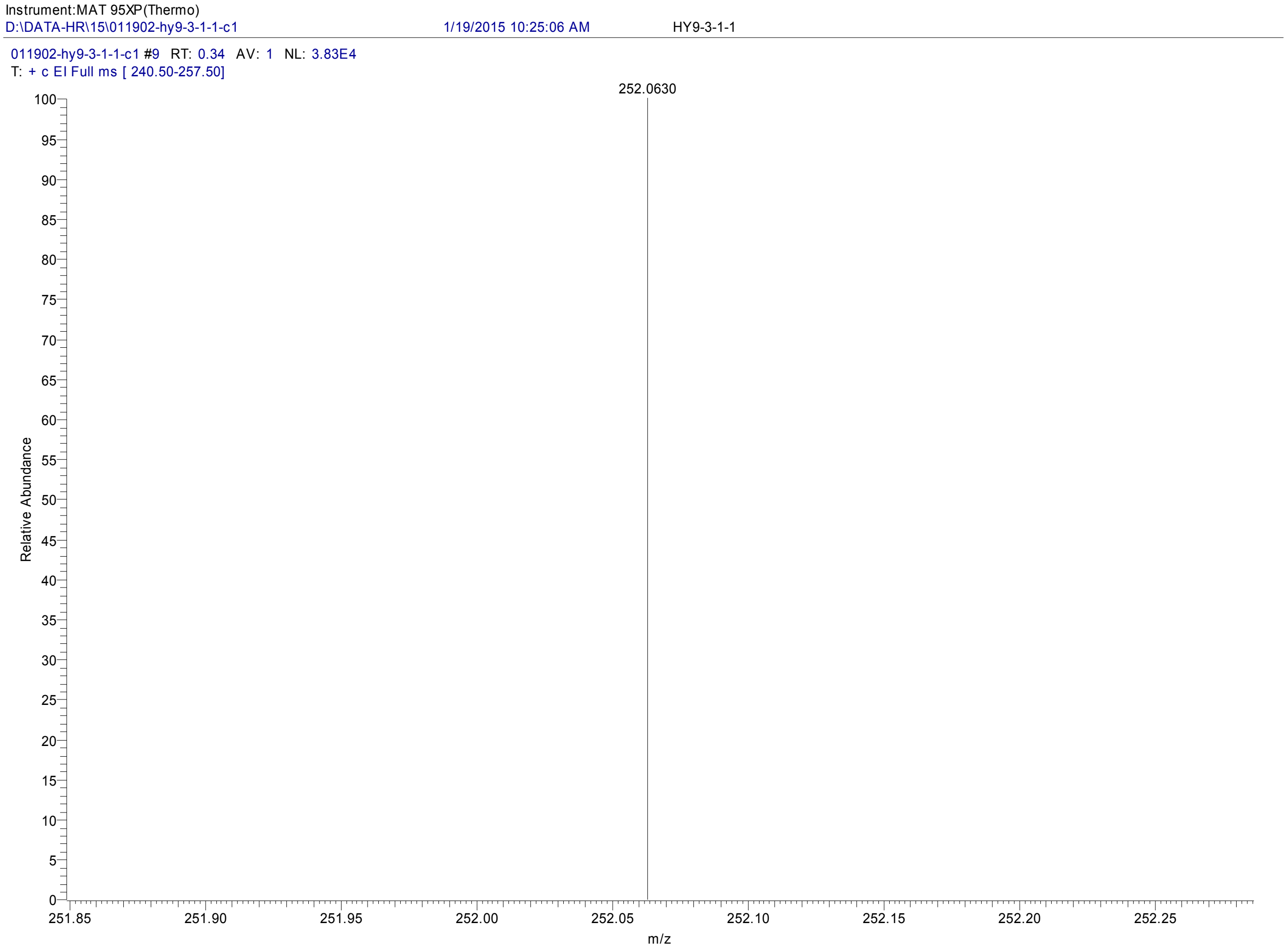

Figure S14. HREIMS spectrum of Compound **3**.

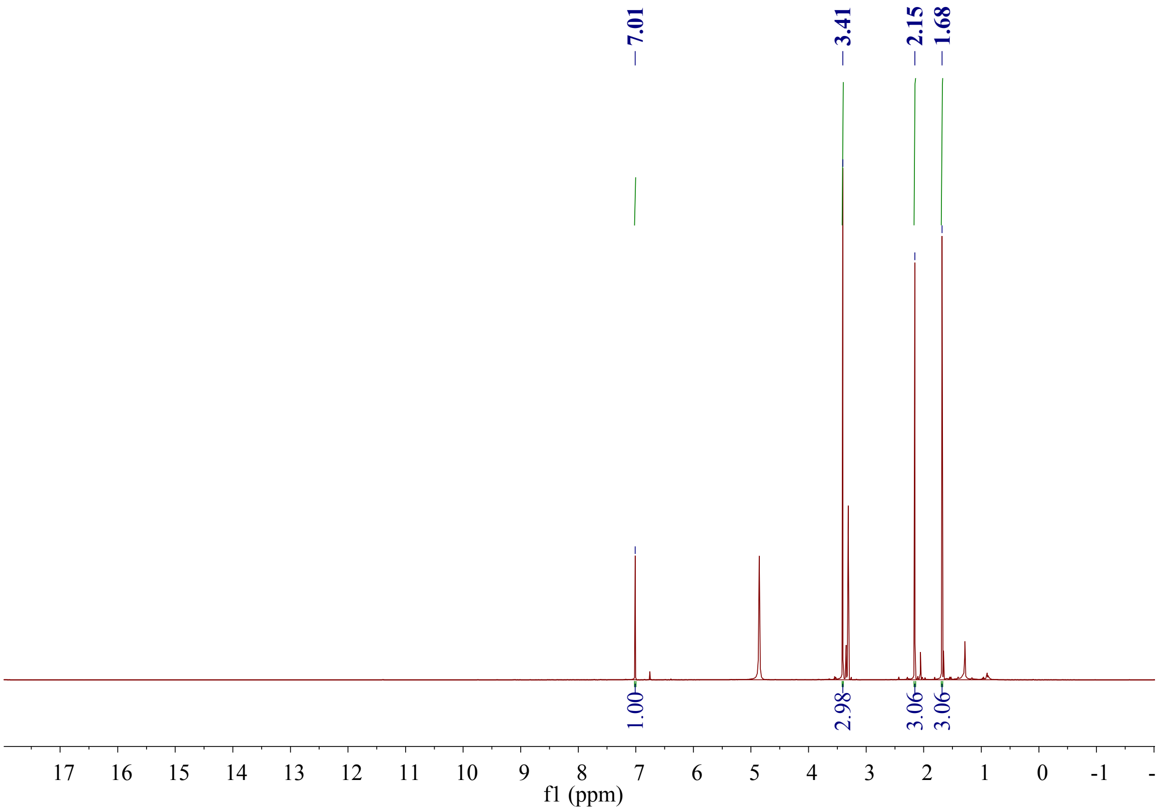

Figure S15. ^{1}H NMR (500 MHz, Methanol-d_4) spectrum of Compound **3**.

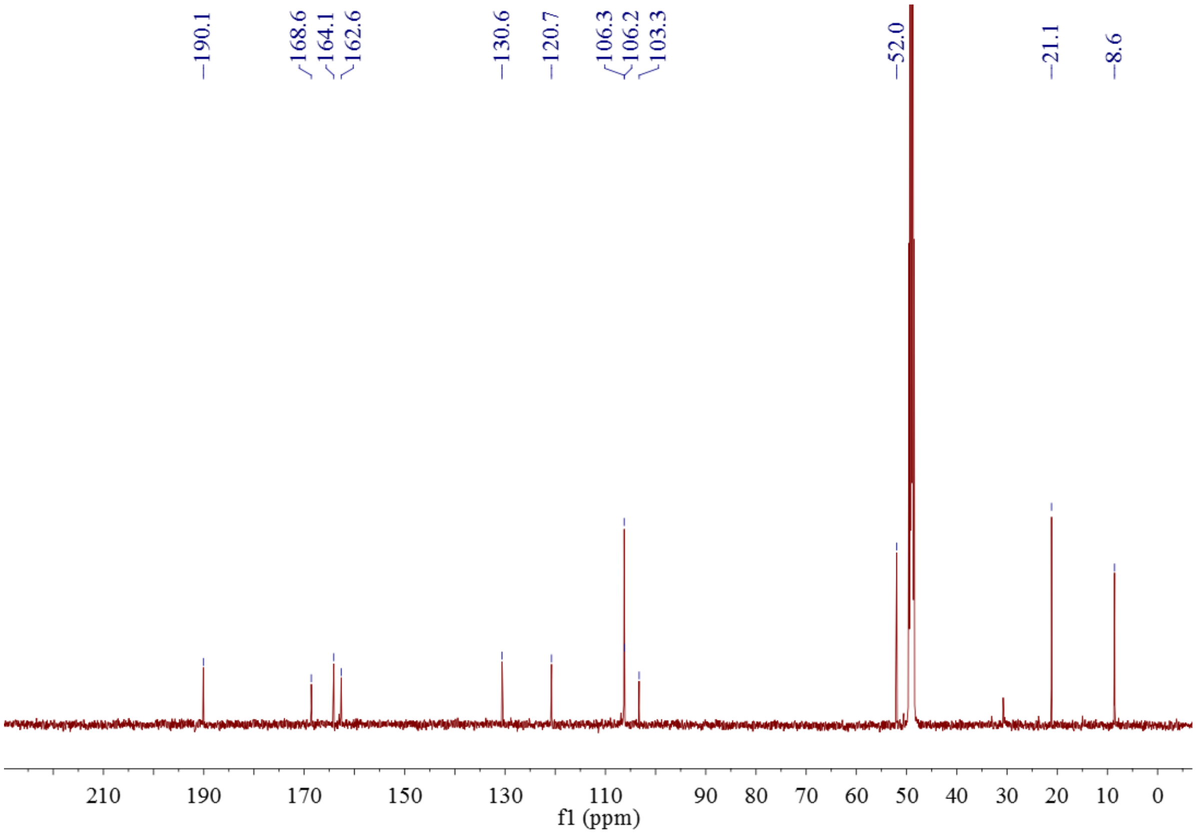

(**A**)

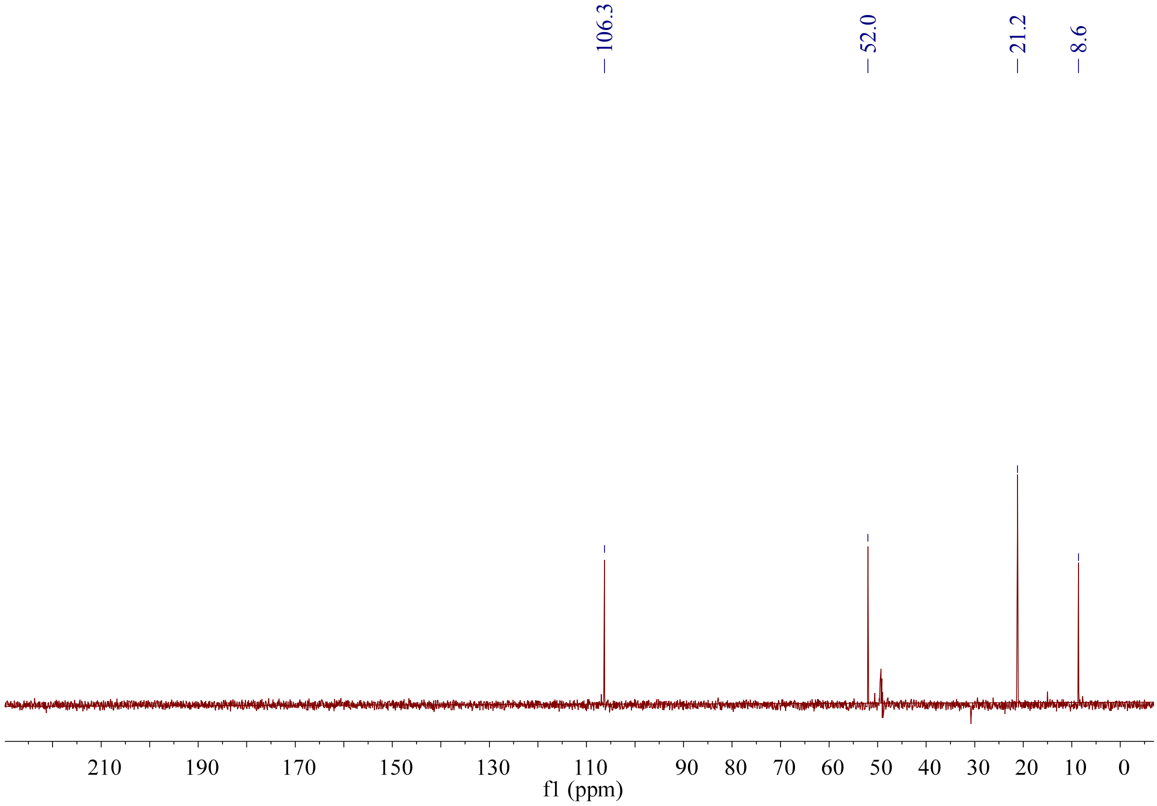

(**B**)

Figure S16. ^{13}C NMR (125 MHz, Methanol-d_4) (**A**) and DEPT spectra of Compound **3** (**B**).

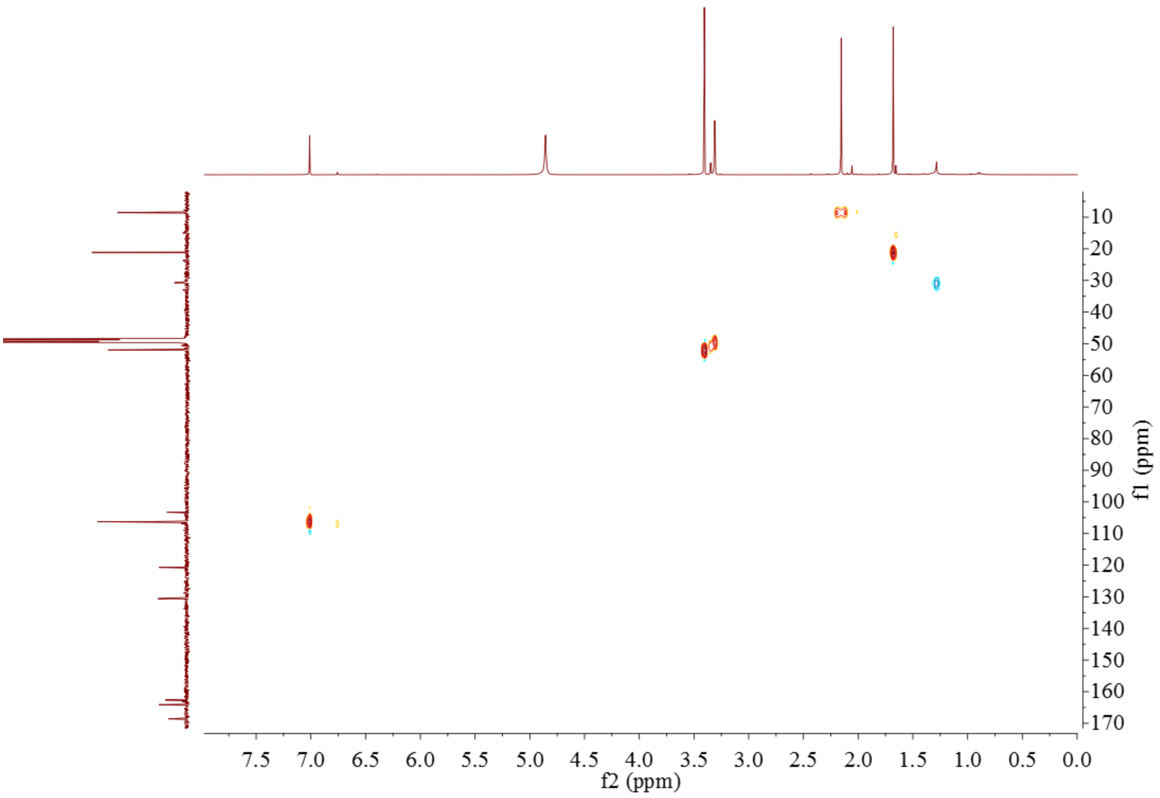

Figure S17. HSQC spectrum of Compound **3**.

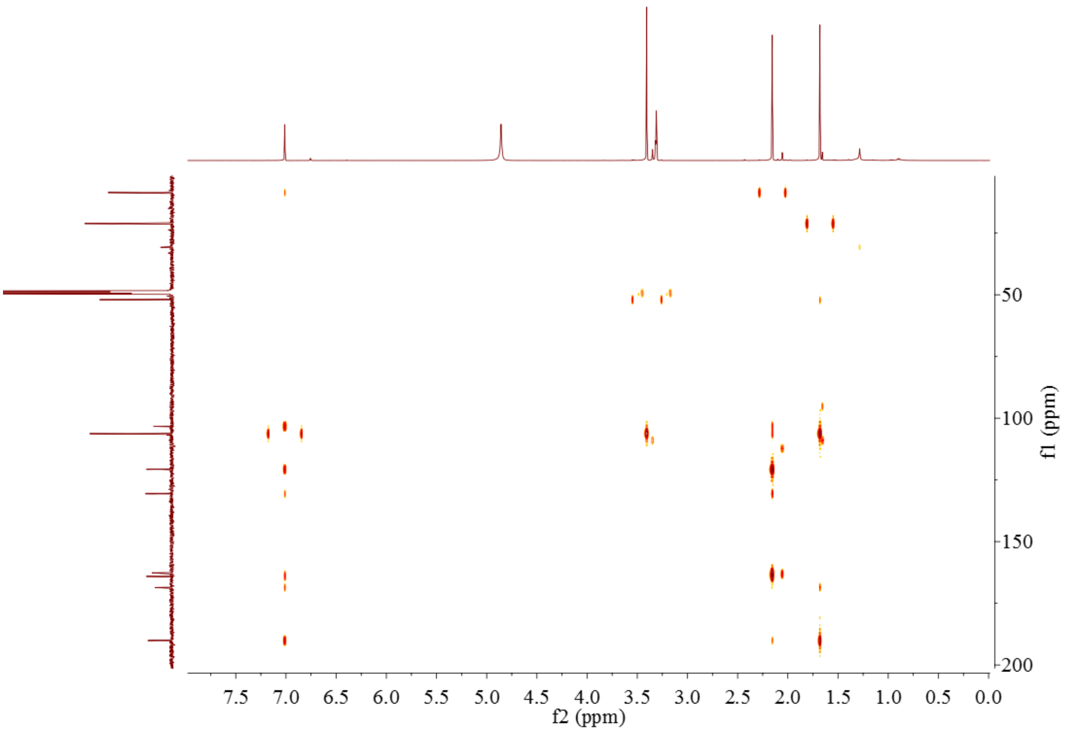

Figure S18. HMBC spectrum of Compound **3**.

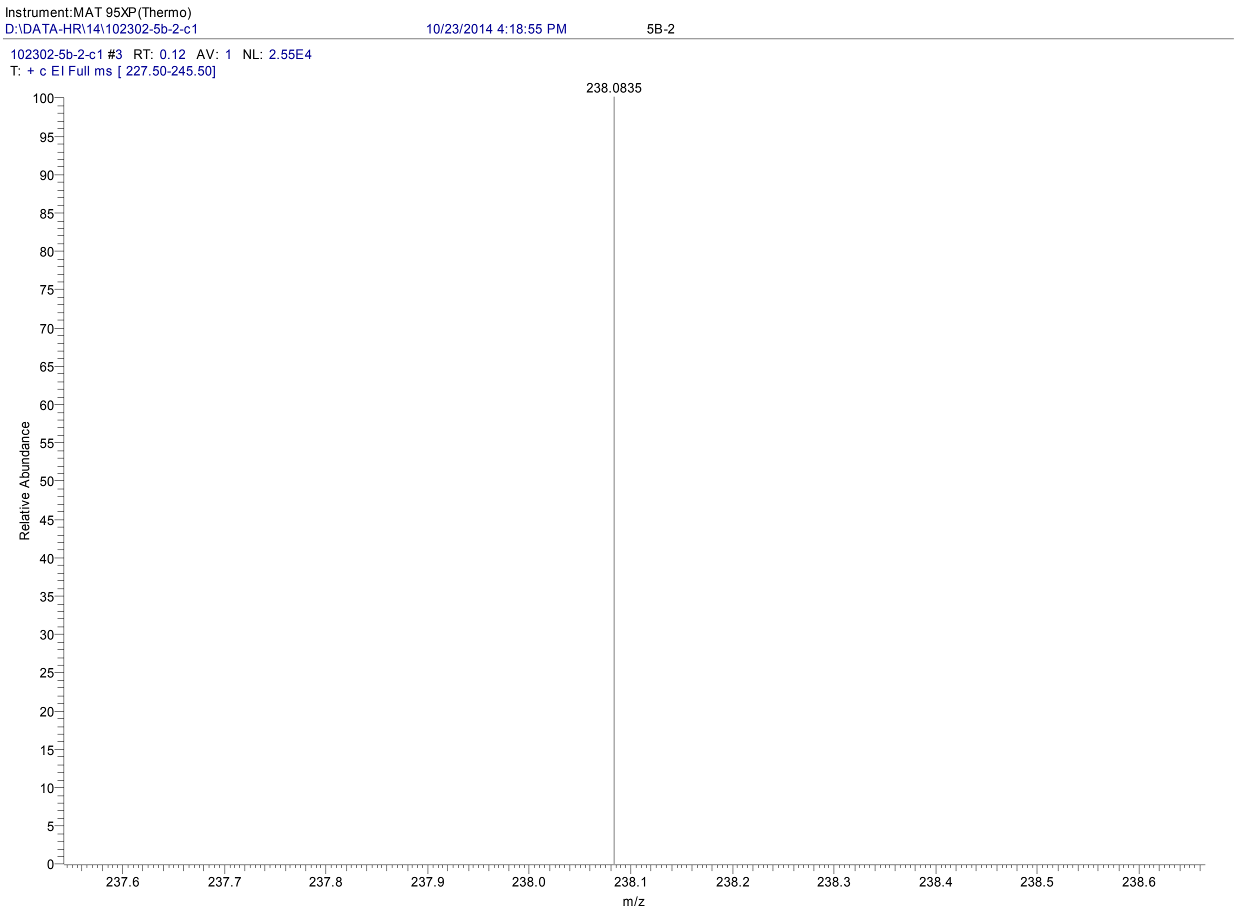

Figure S19. HREIMS spectrum of Compound **4**.

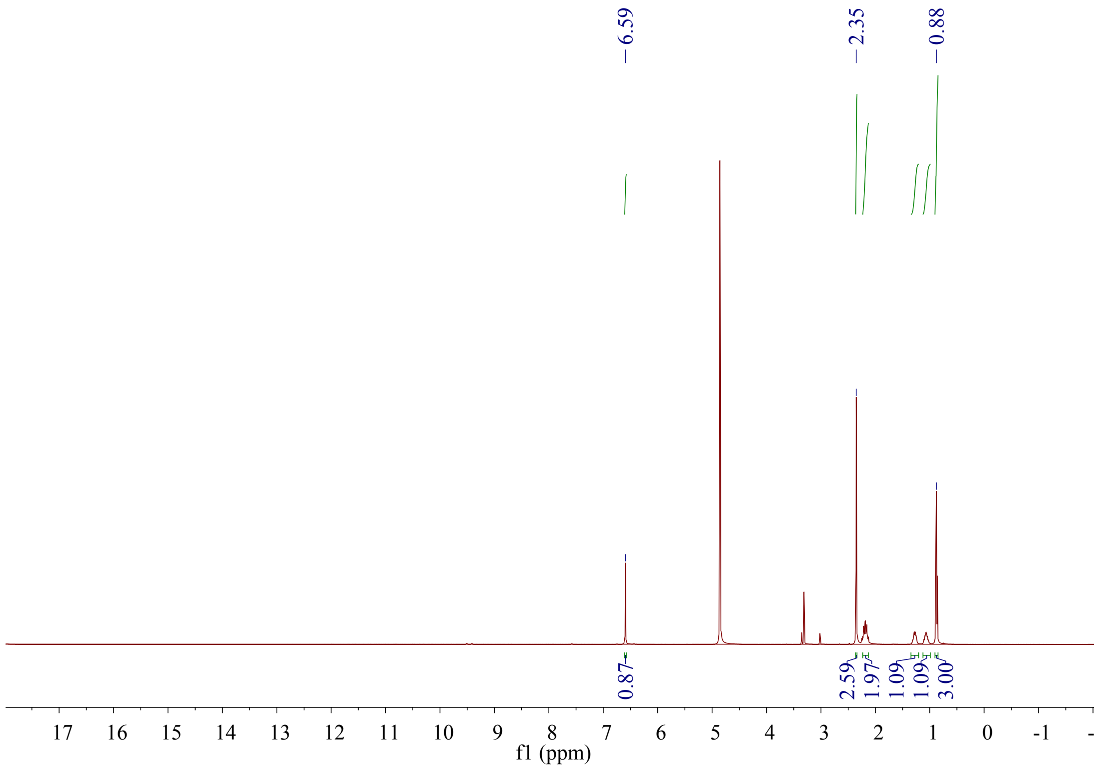

Figure S20. ¹H NMR (500 MHz, Methanol-*d*4) spectrum of Compound **4**.

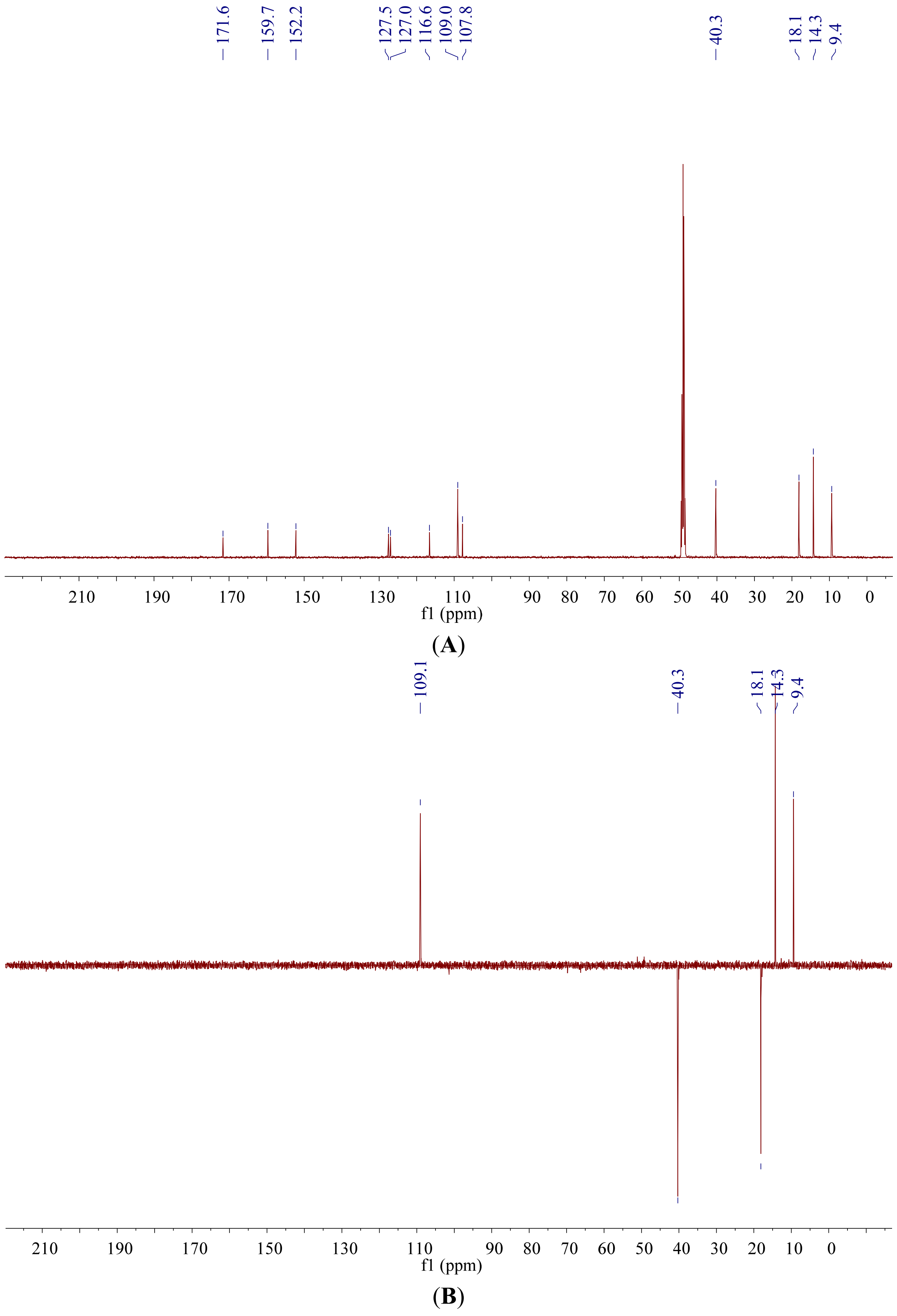

Figure S21. ^{13}C NMR (125 MHz, Methanol-*d*4) (**A**) and DEPT spectra of Compound **4** (**B**).

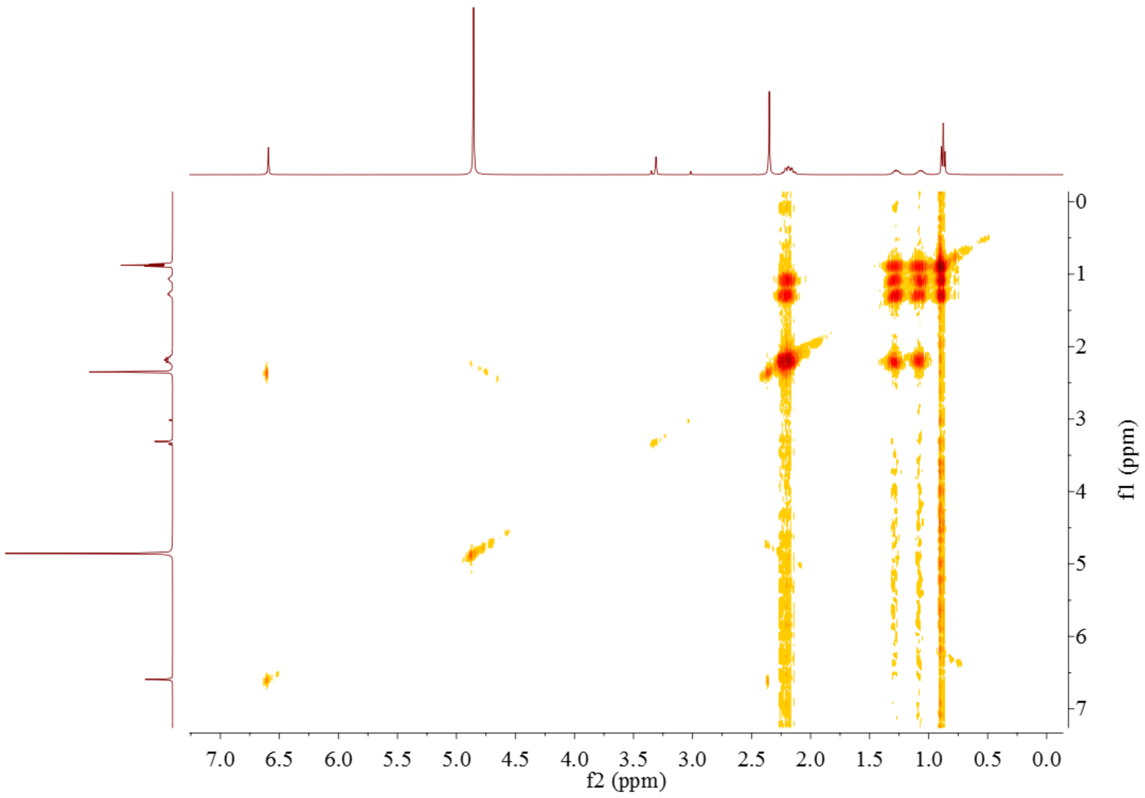

Figure S22. ^{1}H–^{1}H COSY spectrum of Compound **4**.

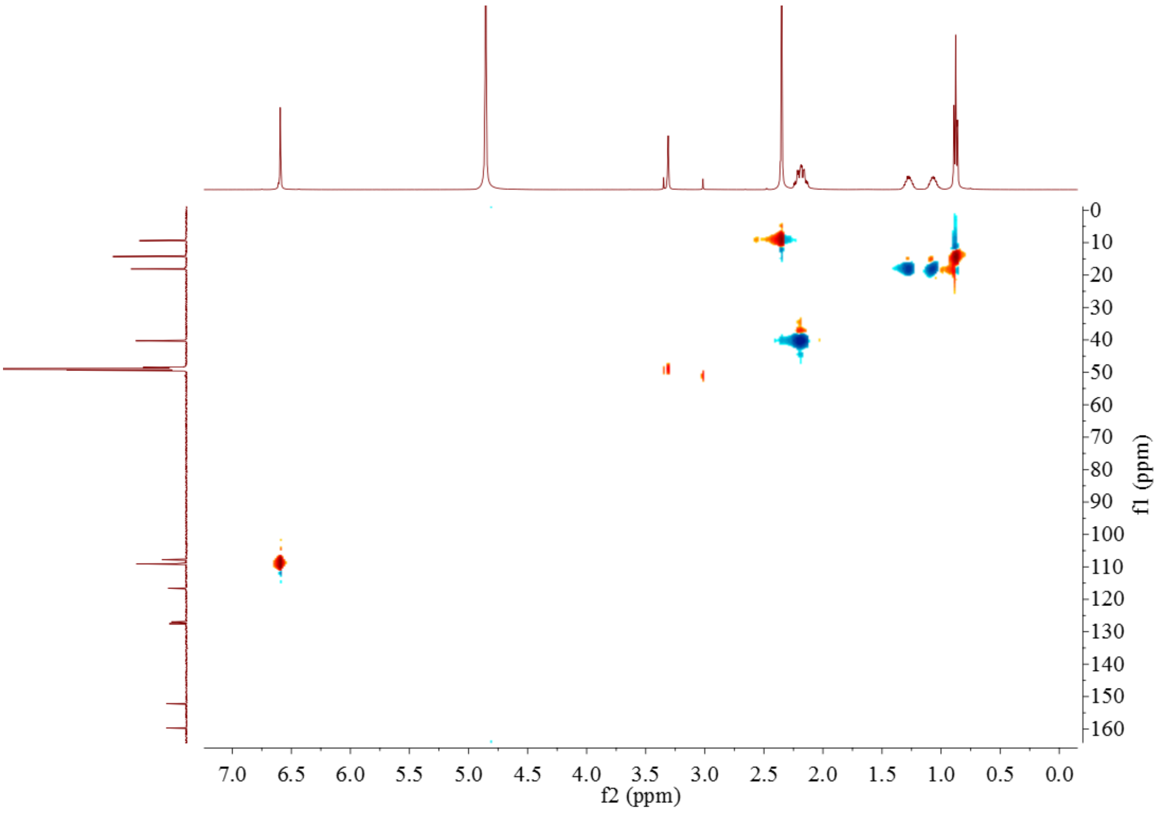

Figure S23. HSQC spectrum of Compound **4**.

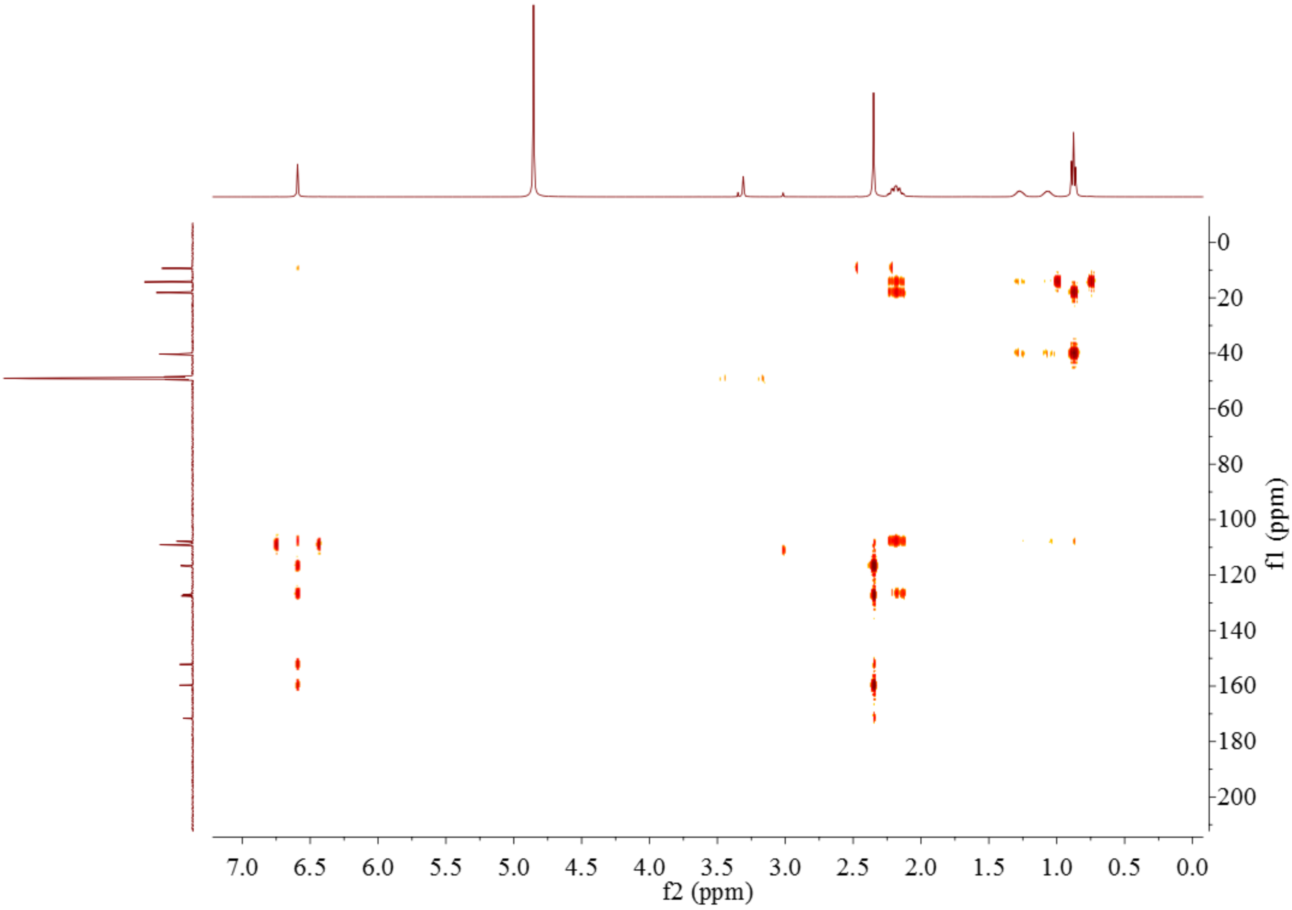

Figure S24. HMBC spectrum of Compound **4**.

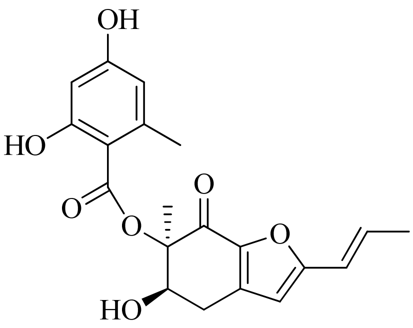

5*R*, 6*R*-1

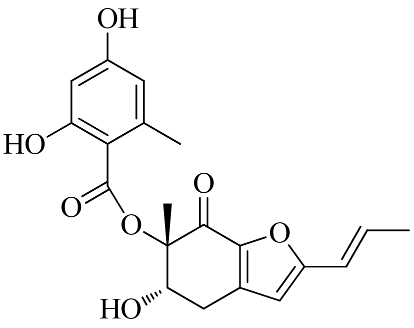

5*S*, 6*S*-1

(A)

Figure S25. *Cont.*

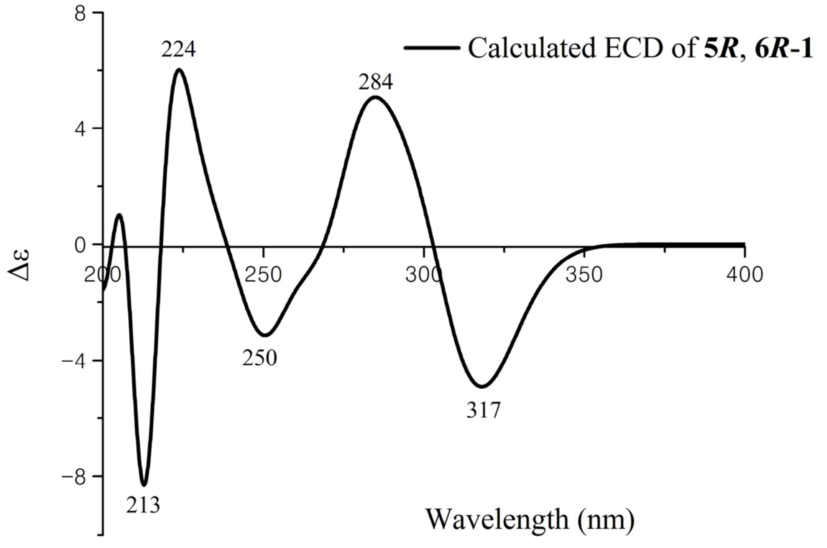

(**B**)

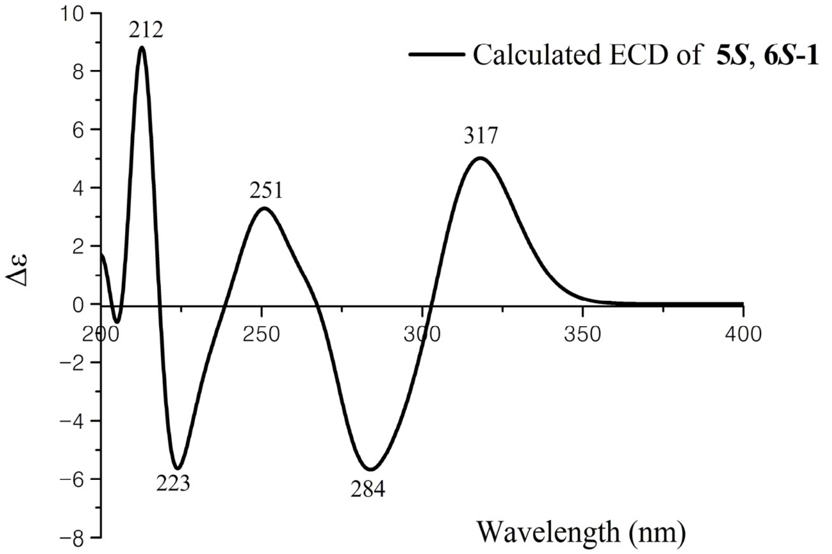

(**C**)

Figure S25. The structures (**A**) and calculated ECD spectra of the two isomers of 5*R*,6*R*-**1** (**B**) and 5*S*,6*S*-**1** (**C**).

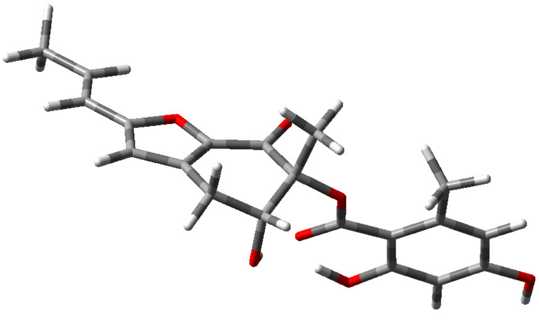

1-1
ΔE = 0 Kcal/mol

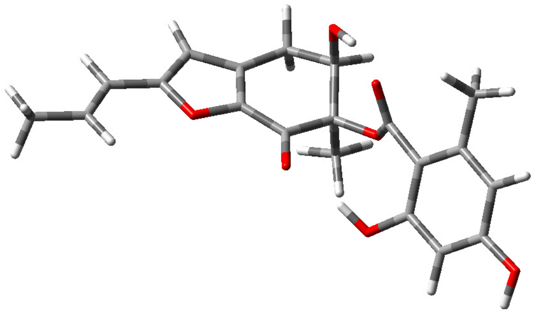

1-2
ΔE = 0.52 Kcal/mol

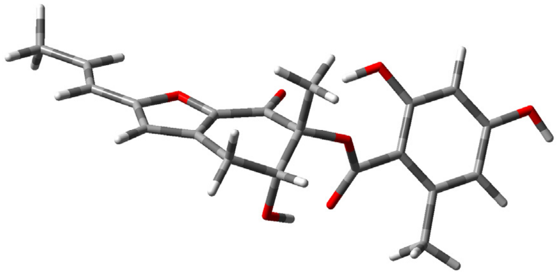

1-3
ΔE = 0.92 Kcal/mol

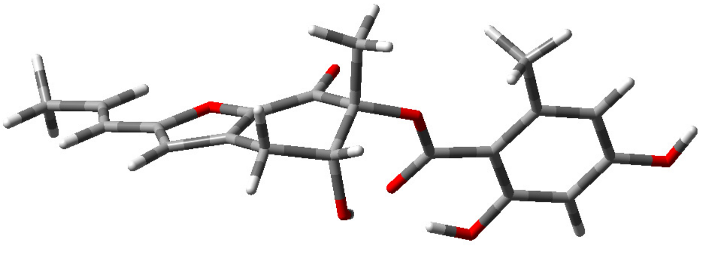

1-4
ΔE = 0.55 Kcal/mol

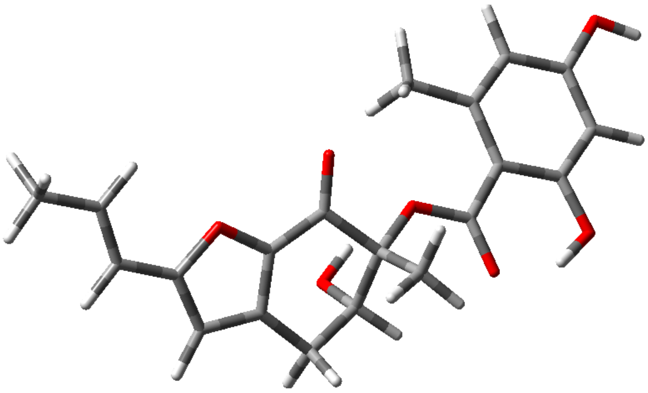

1-5
ΔE = 1.32 Kcal/mol

Figure S26. DFT-optimized low-energy structures (**1-1**–**1-5**) for 5*R*,6*R*.

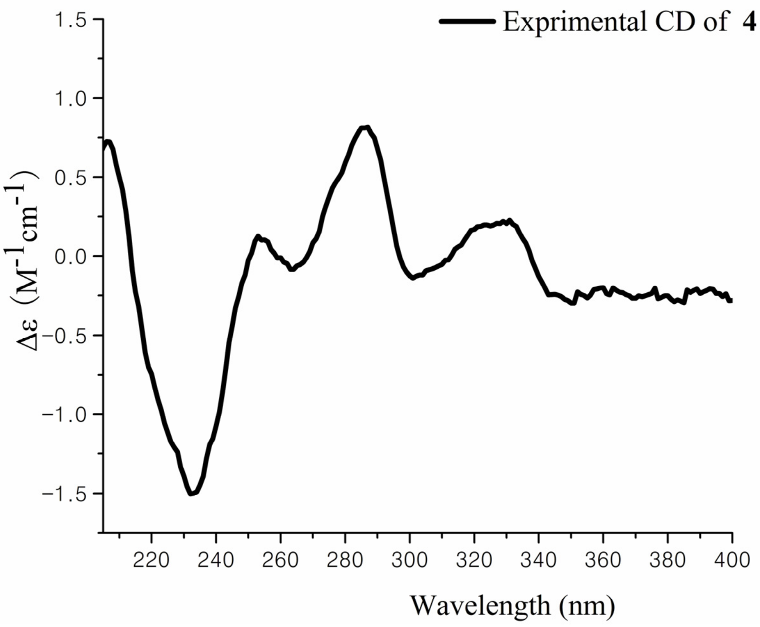

Figure S27. CD spectrum of Compound **4**.

© 2015 by the authors; licensee MDPI, Basel, Switzerland. This article is an open access article distributed under the terms and conditions of the Creative Commons Attribution license (http://creativecommons.org/licenses/by/4.0/).
